# Signal Protein-Derived Peptides as Functional Probes and Regulators of Intracellular Signaling

**DOI:** 10.4061/2011/656051

**Published:** 2011-08-23

**Authors:** Alexander O. Shpakov

**Affiliations:** I.M. Sechenov Institute of Evolutionary Physiology and Biochemistry, Russian Academy of Sciences, Thorez avenue 44, 194223 St. Petersburg, Russia

## Abstract

The functionally important regions of signal proteins participating in their specific interaction and responsible for transduction of hormonal signal into cell are rather short in length, having, as a rule, 8 to 20 amino acid residues. Synthetic peptides corresponding to these regions are able to mimic the activated form of full-size signal protein and to trigger signaling cascades in the absence of hormonal stimulus. They modulate protein-protein interaction and influence the activity of signal proteins followed by changes in their regulatory and catalytic sites. The present review is devoted to the achievements and perspectives of the study of signal protein-derived peptides and to their application as selective and effective regulators of hormonal signaling systems *in vitro* and *in vivo*. Attention is focused on the structure, biological activity, and molecular mechanisms of action of peptides, derivatives of the receptors, G protein *α* subunits, and the enzymes generating second messengers.

## 1. Introduction

The transduction of signals generated by hormones and hormone-like substances of different nature to intracellular effector proteins controlling the fundamental cellular processes requires coordinated activity of many signal proteins, components of a wide spectrum of G protein-coupled and G protein-independent signaling systems, and has several steps in common. The first step is the recognition and specific binding of ligands with extracellular domains of sensors represented by some families of transmembrane proteins, such as the G protein-coupled receptors (GPCRs) seven times penetrating the plasma membrane, the tyrosine kinase receptors having a single transmembrane region (TM) and intracellular domain possessing the intrinsic tyrosine kinase activity, the natriuretic peptide receptors including the membrane-bound guanylyl cyclases, and natriuretic peptide clearance receptor (NPR-C) lacking cyclase activity. The ligand binding is responsible for alteration of conformation of the extracellular regions of receptor and, in the case of GPCRs, for changes of the three-dimensional structure of receptor transmembrane channel (TMC) participating in formation of the ligand-binding site, which starts to transfer the external signal across the plasma membrane and triggers intracellular signaling cascade [1–3]. In the case of G protein-coupled signaling systems the second step of signal transduction is the interaction of intracellular regions of ligand-activated receptor with *α* subunit and/or *βγ* dimer of heterotrimeric G protein in inactive, GDP-bound, state, which induces the GDP/CTP exchange in guanine nucleotide-binding site of G*α* subunit and the dissociation of GTP-bound G*α* subunit from G*βγ* dimer, and the third step is the interaction of GTP-bound G*α* subunit or free G*βγ* dimeric complex with the enzymes, adenylyl cyclase (AC) and phospholipase C (PLC), generating the second messengers, or with the ionic channels, which significantly amplify the initial signal [4–6]. In the activated state the G*α* subunit possesses intrinsic GTPase activity and hydrolyses the bound GTP to GDP, which returns it to the inactive, GDP-bound, state allowing its association with G*βγ* dimer to form G*α* *βγ*-heterotrimeric complex [7–10]. In the case of receptors possessing the tyrosine kinase activity the second and the third steps are characterized by the functional interaction between the regulatory and catalytic domains located within multifunctional intracellular tail of these sensor proteins, which leads to triggering the cascade of phosphorylation-dephosphorylation of downstream regulatory and effector proteins [11–13]. It should be mentioned that the binding of ligand with the extracellular domain of natriuretic peptide receptor NPR-C leads to interaction between its short cytoplasmic region and G proteins; the latter, the same as in the case of GPCR, become activated. It seems quite likely that some tyrosine kinase receptors, the epidermal growth factor (EGF) receptor in particular, have a similar mechanism of action [11, 14, 15]. 

Nowadays it is commonly accepted that the regions of signal proteins participating in their functional interaction and, consequently, responsible for transfer of hormonal signal to cell are rather short and usually contain 8 to 20 amino acid residues [16]. Using different approaches of molecular biology, it is shown that in a majority of cases point mutations in these regions primarily responsible for minimal changes in their conformation drastically influence functional activity of signal proteins and block signal transfer via them. It is not unexpected therefore that the synthetic peptides corresponding to these regions are able to trigger signaling cascades in the absence of hormonal stimulus, modulate protein-protein interaction, and influence the functional activity of signal proteins induced due to changes in their regulatory and catalytic sites. They have been used as important tools in mimicking functional domains of signal proteins, the component of hormonal signaling systems. The present review is devoted to the achievements and perspectives of the study of hormonal signaling systems described in terms of the peptide strategy, a new perspective approach of biochemistry and molecular endocrinology, based on application of synthetic peptides as probes corresponding to functionally important regions of signal proteins, such as receptors of different nature, heterotrimeric G proteins, and the enzymes generating second messengers and responsible for appropriate response of the cell to external signal.

## 2. The Receptors of the Serpentine Type

As a rule, the serpentine type receptors interact with heterotrimeric G proteins and share a highly conserved transmembrane domain consisting of seven hydrophobic TMs (TM1–TM7) and joined by three extracellular loops, three intracellular loops (ICL1, ICL2, and ICL3), extracellular N-terminal domain, and cytoplasmic C-terminal domain (CTD) [17]. The extracellular loops and N-terminal domain form the external surface of TMC participating in ligand-binding function of the receptor, while the ICLs and CTD interact with a number of signal, regulatory and adaptor proteins, including *α* and *βγ* subunits of G protein, RGS-proteins, arrestins, PDZ domain-containing proteins, and G protein-coupled receptor kinases [18, 19].

**Figure 1:**
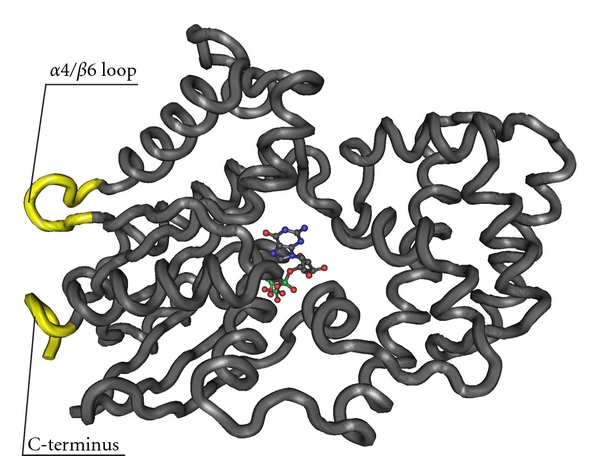
The regions of GTP-bound G*α*_i1_ subunit (*yellow*) responsible for its specific interaction with ligand-activated receptor.

There are numerous data giving evidence that in a majority of GPCRs the membrane-proximal amino- and carboxyl-terminal regions of ICL3 (N- and C-ICL3), TM3/ICL2 interface containing a highly conserved DRY-motif, and the N-terminal region of CTD (N-CTD) forming in some GPCRs an extra, fourth, loop are involved in the binding and activation of G proteins and, thus, are responsible for signal transduction via ligand-activated GPCR to intracellular effector proteins [1, 16, 20–24] (Figure 1). At present the peptides corresponding to these intracellular regions of over 30 GPCRs have been synthesized and their regulatory, and modulatory influence on cellular signaling studied [16, 21, 25–27]. They are successfully used as functional probes to study GPCR-coupled signaling systems, allowing identification of molecular determinants in intracellular domains of GPCRs responsible for their interaction with G proteins and other signal proteins and elucidation of three-dimensional structure and molecular dynamics of intracellular domains of GPCR in inactive, agonist-free, as well as active, agonist-bound, states, revealing the structural-functional organization of oligomeric protein-protein complexes, including receptor molecules, and the role of these complexes in the regulation and control of signal transduction. 

One of the first receptors whose structural organization was studied using the peptide strategy is the light-activated sensor protein rhodopsin belonging to the most ancient family of the serpentine type receptors. Proceeding from the data that synthetic peptides corresponding to ICL2, ICL3, and CTD of rhodopsin significantly inhibit interaction between light-activated receptor and G_t_ protein, transducin, the conclusion was made that the intracellular domains of rhodopsin participate both in binding and in activation of G_t_ protein, which confirms the results of molecular-genetic and crystallographic studies of other authors [28, 29]. It is shown that effective interaction of peptide with G_t_ protein needs involvement of different regions of these domains since combination of two or three peptides, derivatives of ICL2, ICL3, and CTD, as compared to one, gives a 30–50 fold decrease of IC_50_ values for inhibitory effects of peptides on GTPase activity of transducin stimulated by light-activated rhodopsin and leads to complete impairment of coupling between the receptor and G_t_ protein. 

A majority of biologically active GPCR-peptides correspond to ICL3 and ICL2, which is in good agreement with the important role of these loops in productive interaction of GPCR with G proteins and in hormone-induced signal transfer from the receptor ligand-binding site to the enzymes generating second messengers (Table 1). The peptides, derivatives of ICL3 and ICL2 of G_s_-coupled *β*-adrenergic receptors (ARs) and G_i/o_-coupled *α*_2_-AR, selectively bind with G proteins, stimulate their functional activity, trigger signaling cascade in the absence of hormonal stimulus, inhibit the activation of their cognate receptors by selective AR agonists, as it is shown for peptides derived from the sequence 259–273 of *β*_2_-AR ICL3 and the sequence 361–373 of *α*_2_-AR ICL3 [30–37]. In the case of the D_1_- and D_2_-dopamine receptors (DRs), the 5-hydroxytryptamine receptors (5-HTR) of the type 1 and type 6 as well as the m_2_- and m_4_-muscarinic acetylcholine receptors (MChRs), all of which are coupled with G_s_ (D_1_-DR, 5-HT_6_R) or G_i/o_ proteins (D_2_-DR, 5-HT_1A,1B,1D_R, m_2/4_-MChR), and also m_3_-MChR coupled with G_q_ protein it is shown that the peptides, derivatives of the membrane-proximal regions of ICL3, especially C-ICL3, selectively bind and activate G proteins and inhibit their functional interaction with their cognate receptor upon agonist activation [30, 32, 38–44] (Table 1). 

There are many works demonstrating that the peptides corresponding to ICL3 of GPCRs that are activated by the peptide and protein hormones influence GPCR-signaling with high efficiency and selectivity. The ICL3-peptide 237–261 of the *δ*-opioid receptor blocks high-affinity binding of *δ*-agonists, significantly decreases G_i/o_-mediated AC inhibition and PLC activation following the stimulation of the *δ*-opioid receptor, but has no influence on G_s_- and G_q_-coupled signaling cascades regulated via the same receptor [45]. The peptides corresponding to ICL3 of the luteinizing hormone receptor, the follicle-stimulating hormone (FSH) receptor, the relaxin receptor of the type 1 (RXFP1), the glucagon-like peptide-1 receptor (GLP1Rs) and the parathyroid hormone receptor selectively activate distinct types of G proteins and influence signal transduction via their cognate receptors [46–53] (Table 1). 

The cyclic and dimeric forms of GPCR-peptides, as a rule, possess higher activity compared with linear peptides as their three-dimensional structure is more similar to that of the corresponding loop in intact receptor. Among the peptides, derivatives of *α*_2_-AR ICL3, the most potent activator of G_i/o_ proteins is dimeric constrain formed by cross-linked peptides, derivatives of N- and C-ICL3 [31, 54]. A cyclic peptide 225–273 mimicking the full-length ICL-3 of G_s_-coupled V_2_-vasopressin receptor activates G_s_ proteins and significantly inhibits the AC activity stimulated both by vasopressin and by guanine nucleotides, its action on vasopressin-regulated AC system being more potent compared with linear analogs [55]. 

The membrane-proximal N-CTD in some GPCRs forms additional, fourth, intracellular loop and participates in the interaction with GPCR-coupled downstream signal proteins, G proteins in particular. The same refers to peptide 401–417 corresponding to N-CTD of the CB_1_-cannabinoid receptor, which, like ICL3-peptides 301–317 and 329–344, stimulates GTP*γ*S binding of G_i_ proteins and inhibits forskolin-stimulated AC activity [56–58] (Table 1). As for N-CTD-peptide 645–653 of FSH receptor and its short analog, they significantly reduce FSH-stimulated estradiol synthesis in cultured Sertoli cells and increase the GDP/GTP exchange of G proteins in the rat testis membranes [46, 47]. The fact that the peptides corresponding to the membrane-proximal regions of ICL2, ICL3, and CTD of the type 1 angiotensin II receptor activate G proteins and inhibit angiotensin II-induced stimulation of their GTPase activity suggests these regions, the same as rhodopsin, to be involved in the interaction with G proteins [59–62]. Concerning N-formyl peptide receptor (FPR1), peptides 119–133, 122–144, 126–137, and 134–150 corresponding to ICL2, TM3/ICL2, and ICL2/TM4 interfaces as well as CTD-peptides 308–322, 319–340, and 322–336 directly interact with the C-terminal region of G*α*_i/o_, inhibit interaction of G_i/o_ proteins with hormone-activated receptor, and decrease high affinity ligand binding to the receptor, while peptides 210–224, 227–239, and 230–245 corresponding to ICL3, TM5/ICL3, and ICL3/TM6 interfaces have little or no influence on G protein and receptor activities and so do not interfere with their coupling [63, 64]. It is not typical of G protein-binding and -activating surfaces of the receptor to be formed by ICL2 and CTD, so much so as ICL3 is not involved in interaction with G proteins. 

Despite the fact that ICL1 in a majority of GPCRs does not participate in interaction with G proteins, peptide 39–51 corresponding to ICL1 of prostacyclin receptor specifically interacts with the C-terminal region of G*α*_s_ and, thus, activates G_s_ protein and stimulates AC activity. All this indicates an important role of ICL1 of this receptor in G_s_ protein coupling [65, 66] (Table 1). Another example is ICL1 of GLP1R participating, like ICL3, in the interaction with G proteins. The evidence for this stems from the fact that peptide 169–176 corresponding to ICL1 of GLP1R significantly activates G_s_ and G_i_ proteins and influences hormone-stimulated AC activity [53]. 

The action of many GPCR-peptides on G proteins is very selective (Table 1). N-CTD-peptide 401–417 of CB_1_-cannabinoid receptor decreases significantly the interaction of its cognate receptor with G*α*_i3_ subunit, but not with G*α*_i1_ and G*α*_i2_, whereas peptides 301–317 and 329–344 disturb receptor-mediated activation of G*α*_i1_ and G*α*_i2_ subunits, but not G*α*_i3_ [56, 57]. Peptide 483–497 corresponding to C-ICL3 of G_q_-coupled m_3_-MChR at micromolar concentrations selectively activates G_q_ but not G_i2_ proteins [43], while peptide 260–267 corresponding to C-ICL3 of G_s_-coupled D_1_-DR selectively activates G_s_ proteins [38]. Peptides 206–224, 214–231, and 213–225 derived from N-ICL3 of D_2_-DR, 5-HT_1A_R, and 5-HT_1D_R and peptides 382–400 and 285–298 derived from C-ICL3 of m_4_-MChR and 5-HT_1D_R stimulate preferably G_i/o_ proteins and have little or no effect on the other G protein types [30, 32, 40–42]. The N-ICL3-peptide 216–231 of the type 1 angiotensin II receptor coupled with the G_i/o_ and with the G_q/11_ proteins selectively activates purified G_i/o_ proteins, while C-ICL3-peptide 230–241 activates purified G_q/11_ proteins [59, 61]. We have shown that peptides 615–629 and 258–268 corresponding to C-ICL3 of the relaxin receptor RXFP1 and 5-HT_6_R at micromolar concentrations stimulate AC activity and increase the basal level of GTP binding of G_s_ proteins but do not affect the other types of G proteins, in particular the G_i_ proteins associated with AC in the inhibitory manner [39]. The treatment of membrane fraction with pertussis toxin (PT) inactivating G_i_ proteins does not influence the effects of peptides 615–629 and 258–268, whereas the treatment with cholera toxin impairing the function of G_s_ proteins blocks these effects. In the presence of peptide derived from C-terminal segment of the G*α*_s_ subunit the effects of both peptides are significantly decreased. This is due to competition between the full-size G*α*_s_ and C-terminal G*α*_s_-peptide for binding with GPCR-peptide, and is the evidence of participation of G_s_ protein in the mechanism of action of GPCR-peptide. Besides, the peptide corresponding to G*α*_i_ C-terminal segment does not affect the activity of peptides 615–629 and 258–268, derivatives of G_s_-coupled receptors. The C-ICL3-derived peptide 300–316 of G_i_-coupled 5-HT_1B_R increases GTP binding activity of G_i_ protein but does not affect the other G proteins. This effect is blocked by PT treatment and reduced in the presence of C-terminal G*α*_i_-peptide [39]. 

The G protein-interacting regions of GPCRs are usually positively charged and contain BBXXB, BBXB, and the related motifs, where B is a basic amino acid residue (Arg or Lys) and X is another residue [30, 90, 91], specifically interact with the negatively charged C-terminal region of G*α* subunits that is of prime importance for recognition and effective interaction of different types of G proteins with ligand-activated GPCR [42, 92–94]. In addition to the extreme C-terminus of G*α*, the *α*4 helix and the *α*4/*β*6 loop rich in negatively charged amino acid residues also participate in a receptor-G protein coupling [32]. As a result, the presence of BBXXB and the related motifs in GPCR-peptides, derivatives of receptor intracellular domains, is a very important structural feature required for their effective and selective interaction with G proteins. The substitution of the positively charged Lys^221^ and Arg^222^ in C-terminal segment of N-ICL3-peptide 208–226 of G_i/o_-coupled D_2_-DR by alanine residues leads to a 3-fold reduction in its affinity for G*α*_i/o_ [42]. Consistent with that, mutant D_2_-DR with the substitution of segment RRRR^217-220^ in N-ICL3 by AAAA is not capable of effective interaction with G_i/o_ proteins and is insensitive to agonist activation. These findings support the view that charge-charge complementation has a very important role in receptor-G*α* coupling [95, 96]. 

The peptide strategy makes it possible to produce analogs of GPCR-peptides with a higher biological activity and to deliver them to the intracellular effector proteins of the target cells, which is very important for their application as a new generation of highly effective and selective GPCR-based drugs. The most perspective way to achieve this goal is modification of GPCR-peptides using either hydrophobic radicals, for example, C_12_–C_18_ acyl or steroid group, or hydrophobic TM fragment; they mimic the receptor TM, maintain the peptide conformation close to that in native receptor, and, finally, allow the transfer of peptide across the plasma membrane. Such cell-penetrating lipidated peptides are designated as pepducins. The latter corresponding to ICLs of the protease-activated receptors (PAR1, PAR2, PAR4), the chemokine receptors (CXCR1, CXCR2, CXCR4, CCR5), the sphingosine-1-phosphate (S1P) receptor, the relaxin receptor RXFP1, *α*_1B_-AR, 5-HT_1B_R, and 5-HT_6_R function as intracellular agonists or antagonists of their cognate receptors and their action is realized at the stage of specific interaction between the receptor and G protein on the intracellular plasma membrane surface [39, 49, 50, 97–102]. Pepducins can be used to control platelet-dependent hemostasis and thrombosis, tumor growth, invasion, angiogenesis, as well as to prevent some inflammatory diseases [26, 27, 100, 103–109]. 

With respect to the specificity and efficiency of action on their cognate receptors, pepducins significantly surpass unmodified analogs, which makes them very perspective selective regulators of hormonal signaling systems [39, 50, 98–100, 105, 110, 111]. Peptide Pal-RCLSSSAVANRS^295-306^ (P1pal-12) corresponding to N-ICL3 of G_q/11_- and G_i_-coupled PAR1 functions as a selective PAR1 antagonist and inhibits the increase in inositol triphosphate production and concentration of intracellular Ca^2+^ induced by agonist-activated receptor, significantly decreases platelet aggregation in response to PAR1 agonist SFLLRN and the vasorelaxation caused by other PAR1 agonist TFLLR-amide in the rat aorta, but does not affect the vasorelaxation caused by PAR2 agonist SLIGRL-amide, neither does it inhibit PAR4-dependent platelet aggregation [98, 112]. This peptide has no effect on the aggregation in response to agonists of the thromboxane, ADP, collagen, and GPIb/IX/V receptors the same as the lack of effect on the response to interleukin-8, stromal-derived factor 1*α*, S1P, monocyte chemotactic protein-1, RANTES (Regulated on Activation Normal T cell ExpreSsed) in endothelial cells, or the migration of recombinantly transfected HEK293 cells to ligands of PAR2, PAR4, CXCR1, CXCR2, CCR5, or S1P receptors [98, 105]. Peptide Pal-RCLSSSAVANRSKKSRALF^295-313^ (P1pal-19), a derivative of PAR1 ICL3, possesses the activity of PAR1 agonist and, like selective PAR1 agonists TFLLR and SFLLRN, stimulates the G_q_ and G_i/o_ proteins and PLC*β* activities, produces endothelial NO-dependent relaxation in the rat aorta, and promotes prostaglandin E_2_ release in the human lung epithelial cells and the rat gastric mucosal epithelial cells [97, 98, 112]. The action of P1pal-19 is very specific to PAR1 and its influence is drastically reduced or completely blocked in cultured cells and the tissues where its cognate receptor is absent or mutant form of PAR1 is expressed. 

The ICL3-peptide Pal-SGRRYGHALR^274-283^ (P4pal-10) of PAR4 functions as an antagonist of its cognate receptor and a partial antagonist of PAR1. It completely inhibits PLC*β* stimulation and Ca^2+^ response induced by PAR4 agonist AYPGKF-amide and decreases the analogous effects of PAR1 agonist SFLLRN-amide by 36%. P4pal-10 completely blocks platelet aggregation induced by PAR4 agonist and partially inhibits PAR1 agonist-induced aggregation, whereas it has no effect on the aggregation of platelets induced by a series of agonists of the GPCRs which do not belong to the PAR family, neither on the migration of human neutrophils to interferon-*γ*-inducible protein-10, stromal-derived factor 1*α*, and S1P but completely blocks migration to thrombin activating both PAR1 and PAR4 [98, 111]. ICL1-peptide Pal-ATGAPRLPST^103-112^ (P4pal-i1) of PAR4 is more selective compared with peptide P4pal-10. It completely blocks the chemotactic response realized via PAR4 and prevents platelet aggregation triggered by PAR4 agonist AYPGKF-amide but does not inhibit the chemotactic response of PAR1 and does not affect platelet aggregation induced by PAR1 agonist SFLLRN-amide [106, 113]. When P4pal-i1 is combined with thrombin inhibitor bivalirudin, inhibition of human platelet aggregation and suppression of arterial thrombosis in guinea pigs increases to a much higher degree than with bivalirudin alone [106]. The data concerning thrombolytic action of peptides P4pal-10 and P4pal-i1 and ICL-peptides of other PARs show that on their basis it is possible to have receptor-selective drugs for antiplatelet therapy [114]. 

Pepducins have been used to study the role of the chemokine receptors in sepsis and systemic inflammation and are found to improve survival and prevent systemic inflammation and overactivation of the coagulation system in sepsis. Pepducins corresponding to ICL1 and ICL3 of the chemokine receptors CXCR1 and CXCR2 function as selective antagonists of these receptors and inhibit G_s_-mediated effects of interleukin-8, a ligand for these receptors, neutrophil chemotaxis toward interleukin-8 in particular, as well as reverse disseminated intravascular coagulation and liver failure in septic mice and prevent the lethal sequel of sepsis. At the same time, pepducins corresponding to ICL1 and ICL2 of the chemokine receptor CXCR4 selectively interact with their cognate receptors and cause a massive leukocytosis consistent with the role of CXCR4 in stromal-derived factor 1*α* neutrophil homeostasis, while the pepducins do not affect interleukin-8-mediated signaling pathways and have no effect on survival [100, 105]. 

We showed that peptide QVKKE(Nle)ILAKR^619-629^K(Pal) corresponding to C-ICL3 of the G_s_-coupled relaxin receptor RXFP1 stimulates AC activity and G_s_ protein GTP binding and inhibits regulatory effects of relaxin on the AC system in the myocardium and brain, which raises no doubt due to a high level of expression of its cognate receptor, but is ineffective in the skeletal muscles where there are no receptors [49, 50]. Palmitoylated peptide KHSRKALKASL^258-268^K(Pal)A corresponding to C-ICL3 of 5-HT_6_R is effective in the brain rich in 5-HT_6_R, but not in respect to the AC system in the myocardium and testes where these receptors are absent or expressed very little [39]. 

Alongside with high selectivity, pepducins are more efficient compared with their unmodified analogs. Actually, RXFP1-peptide 619–629-K(Pal) stimulates relaxin-sensitive AC system with greater efficiency compared with 15-mer peptide 615–629 lacking fatty acid radical, while unmodified 11-mer peptide is not active in this case [50]. Palmitoylated 5-HT_6_R-peptide 258–268 has a stronger influence on G_s_ proteins and AC than its unmodified analog [39]. PAR1-peptide 295–313 lacking hydrophobic radical does not stimulate Ca^2+^ fluxes while its palmitoylated analog causes a rapid Ca^2+^ transient [97]. 

These data give grounds to make a conclusion that modification by hydrophobic radicals is one of the most perspective approaches to enhance both the selectivity and efficiency of GPCR-peptides as regulators of hormonal signaling systems. The main reason for high biological activity of pepducins is the ability of their hydrophobic radical to partition into the plasma membrane and to flip across lipid bilayer, thus shuttling the attached peptide to the intracellular surface of the membrane and mediating the effective interaction of peptide portion of pepducin with the complementary regions of the GPCR or G proteins [115].

## 3. The Receptors with Tyrosine Kinase Activity

In the 1990s the peptide strategy found wide application with a view to identify signal-protein-interacting regions in two best known receptors possessing tyrosine kinase activity, the insulin receptor (IR) and the EGF receptor. The IR belongs to the family of transmembrane glycoprotein receptors activated by insulin, insulin-like growth factor-1 and other insulin-related peptides, and is composed of two 135-kDa extracellular *α* subunits which bind the hormone and two 95-kDa transmembrane *β* subunits with intrinsic tyrosine kinase activity. The main substrates of insulin-stimulated phosphorylated IR are IRS and Shc proteins, but the data are available suggesting that IR is also able to interact with heterotrimeric G proteins and, thus, regulate G protein-dependent signaling cascades [11, 15, 116]. 

The first data concerning the regions capable of specifically interacting with G proteins appeared in the early 1990s and were obtained with three synthetic peptides, 1039–1061, 1135–1156, and 1319–1333, corresponding to the tyrosine kinase domain (the first two) and the C-terminal domain (the third) located in the cytoplasmic tail of IR *β* subunit [117]. They contain positively charged BBXXB and the related motifs typical of G protein-activating sequence of GPCRs. Peptide 1039–1061 induces a dose-dependent stimulation of GTP-binding and GTPase activities of G_s_ proteins, while peptide 1325–1345 containing a site for tyrosine phosphorylation located in the C-terminal domain of IR activates the G_i/o_ proteins [117], and peptide 1135–1156 containing tyrosine residues critical for IR tyrosine kinase activity stimulates GTP-binding of unusual 67 kDa G protein involved in the insulin signal transduction [118]. The G_i_-activating effect of C-terminal peptide 1325–1345 is supported by the data obtained later on direct interaction between insulin-activated IR with G_i2_ protein and the blocking of this interaction by PT [116, 119]. 

To identify IR regions participating in phosphorylation-dephosphorylation cascade, a series of peptides containing the most important tyrosine residues, the targets of tyrosine phosphorylation, and their derivatives with modified tyrosines were synthesized and studied (Table 1). A tris-sulfotyrosyl peptide 1142–1153 corresponding to the major site of autophosphorylation inhibits dephosphorylation of the IR in CHO cells overexpressing the human IR [67, 120]. An N-stearyl derivative of this peptide causes a several-fold increase in insulin- and vanadate-stimulated phosphorylation of IR and leads to a significant enhancement of insulin-induced stimulation of phosphatidylinositol 3-kinase (PI3-K) and mitogen-activated protein kinase (MAPK) activities but has no effect on the basal phosphorylation of IR. Maximal stimulation is observed with 50 *μ*M of N-stearyl peptide 1142–1153. Peptide 1293–1307 corresponding to C-terminal domain of IR enhances insulin-stimulated receptor autophosphorylation but has no detectable effects on the basal phosphorylation of IR and on receptor dephosphorylation [68]. A stearyl analog of peptide 1293–1307 also enhances insulin-stimulated autophosphorylation and, additionally, induces a 2-3-fold increase in both the activity of insulin-stimulated PI3-K, and the level of tyrosine phosphorylation of MAPK in response to insulin. The most pronounced influence of N-stearyl derivatives of peptides 1142–1153 and 1293–1307 on insulin-induced IR autophosphorylation and function, compared with unmodified analogs, suggests that the intrinsic activity of IR undergoes modulation, depending on the presence of a fatty acid-acylated receptor fragment in cells. The expression of a minigene encoding 23-mer peptide 1292–1315, the longest analog of peptide 1293–1307, has no effect on the basal receptor tyrosine phosphorylation level but leads to an increase in insulin-activated IR autophosphorylation that in minigene-transfected CHO cells is about twice as high as in control cells [121]. The stimulating effects of insulin on PI3-K and MAPK activities and thymidime incorporation into DNA are also significantly elevated in cells expressing the minigene. These data indicate that peptides 1142–1153 and 1293–1307 and minigene-expressed peptide 1292–1315 bind to the regions in IR *β* subunit responsible for the intrinsic activity of the receptor and functional coupling with downstream signaling proteins, such as IRS and Shc proteins, PI3-K and MAPK. The action of the peptides and their lipophilic derivatives is highly specific since the latter are not capable of inhibiting dephosphorylation of EGF receptor and do not influence ligand-induced phosphorylation of both EGF receptor and insulin-like growth factor-1 receptor which functionally and structurally resembles IR [67, 68, 121]. 

To identify the regions participating in IR tyrosine kinase function, Van Obbergen and colleagues used another approach based on antipeptide antibodies directed against synthetic peptides corresponding to the regions localized in the C-terminal domain of IR, such as 1270–1280, 1294–1317, and 1309–1326 [122–124]. Antipeptide IgG recognizing acid-rich sequence 1270–1280 inhibits both receptor autophosphorylation, at least on Tyr^1146^, Tyr^1150^, and Tyr^1151^, the main targets of IR tyrosine kinase, and the receptor-induced phosphorylation of poly(Glu^80^Tyr^20^) and a peptide corresponding to the region 1142–1158 [123]. The antipeptide antibody directed to the region 1294–1317 inhibits phosphorylation of poly(Glu^80^Tyr^20^) and synthetic peptides corresponding to the IR autophosphorylation sites but does not inhibit receptor autophosphorylation. In addition, it decreases receptor-mediated phosphorylation of Src homology/collagen and of IRS-1 protein by 52 and 30%, respectively [124]. These data give strong evidence that the C-terminal domain of IR interacts with signal proteins participating in the regulation and modulation of tyrosine kinase activity of the receptor. 

The EGF receptor one time penetrates the membrane and contains a large extracellular domain that consists of two ligand-binding and two cysteine-rich subdomains and the intracellular tyrosine kinase domain. The binding of EGF receptor to EGF causes receptor dimerization and induces phosphorylation of tyrosine residues in tyrosine kinase domain located in the intracellular tail of receptor, which recruits many signaling proteins, such as PI3-K, PLC-*γ*, PLD, Shc, and Grb2 proteins, phosphotyrosine phosphatase 1, and others, and transduces hormonal signal to different intracellular effector proteins and systems [125]. In 1995 Sun and colleagues revealed that EGF receptor-derived peptide is able to stimulate G proteins [69] (Table 1). This finding coincides very well with the data obtained in the recent years about the direct interaction of different types of G proteins with EGF receptor and their role in EGF-mediated signaling [11, 126, 127]. The synthetic peptide 646–658 corresponding to the membrane-proximal segment of the juxtamembrane domain located in the cytoplasmic tail of EGF receptor stimulates GTP-binding and GTPase activities of G_s_ protein coupled to this receptor. In addition, it increases the G_i_ protein GTPase activity without affecting the GTP-binding activity, which suggests that the juxtamembrane domain is able to stimulate G_s_ proteins and inactivate G_i_ proteins. Peptide with phosphorylated residue Thr^654^, the main target for protein kinase C (PKC), does not affect G_s_ protein and retains the ability to stimulate the GTPase activity of G_i_ protein. The incorporation of negatively charged phosphate group decreases the net positive charge of peptide and, in addition, destabilizes its helical conformation, which is functionally important for effective interaction of peptide with the G*α*_s_ subunit, and, hence, blocks the activation of G_s_ protein by the phosphorylated peptide. It is supposed that in the case of G_i_ protein the phosphorylated peptide induces the dissociation of the heterotrimeric complex to give monomeric G*α*_i_ subunit and, thus, stimulates its GTPase activity. These data speak in favor of the fact that phosphorylation of tyrosine residues in EGF receptor, as well as of serine and threonine residues in GPCRs, affects selectively their coupling with different types of G proteins, G_s_ and G_i_ proteins in particular. It was shown, in addition, that peptide 646–658 with the reverse sequence (RRLTRKRVIERRR) also activates G_s_ protein, which indicates that orientation of peptide 646–658 is of no importance for its interaction with G protein and the main role in this process is given to the secondary structure of peptide (*α* helix is optimal) and spatial distribution of positively charged amino acids [69]. As has been shown recently using the other biochemical approaches, G*α*_i1_ and G*α*_i3_ form the complex with ligand-activated EGF receptor and are required for phosphorylation of Grb2-associated binding protein 1 and its subsequent interaction with p85 subunit of PI3-K in response to EGF, whereas the coupling between EGF receptor and G_s_ protein leads to activation of cAMP-dependent signaling pathways [11, 127]. Thus, the peptide strategy came to be a starting point for further investigations in which the role of G proteins in the tyrosine kinase receptor-mediated signaling has been established. 

A new era of application of the peptide strategy in the study of structural-functional organization of the tyrosine kinase receptors is connected with quantitative proteomics using pull-down experiments with pairs of phosphorylated and nonphosphorylated synthetic peptides, corresponding to phosphotyrosine-containing sites in the intracellular domains of these receptors [13, 128, 129]. The receptors possessing tyrosine kinase activity have, as a rule, several docking sites for each of the interacting proteins-partners. The EGF receptor and the related to it ErbB4 receptor have, for example, four to seven docking sites for Grb2 protein three to four sites for Shc protein, while ErbB3 receptor lacking the functional kinase domain has a large number of binding sites for PI3-K. Using a series of synthetic peptides corresponding to tyrosine phosphorylation sites of EGF and ErbB4 receptors, the new recognition motifs for Shc protein were detected. It was shown that the STAT5 protein, a latent cytoplasmic transcription factor, is a new partner of both receptors; it specifically interacts with Tyr^978^- and Tyr^998^-containing sites of EGF receptor and with Tyr^984^-containing site of ErbB4 receptor. It should be pointed out that both phosphorylated and nonphosphorylated forms of peptides are used to study the role of tyrosine phosphorylation for effective and selective interaction of these receptors with their partners [129]. The assay based on the interaction between receptor-derived phosphotyrosine-containing peptides and proteins-partners is extremely specific and capable of conforming the binding motifs identified so far and detecting new target sequences, which is confirmed by the fact that all identified binding partners contain either SH2- or PTB-domains specifically interacting with phosphotyrosine-containing motifs, and phosphotyrosines not expected to have binding partners do not show any proteins with significant ratios. This assay is very sensitive, since the peptide-protein interaction screen is validated for binding constants as low as at least 5 *μ*M, but it is likely to work with much lower affinity interactions [128, 129].

## 4. Natriuretic Peptide Clearance Receptor

The natriuretic peptide receptor of C-type (NPR-C) belongs to natriuretic peptide receptors but lacks gyanylyl cyclase catalytic domain. It is a disulfide-linked homodimer having a single TM, a large extracellular domain of 440 amino acids, and a short 37-amino acid cytoplasmic tail. The binding of the NPR-C with atrial natriuretic peptide (ANP) and its several fragments induces the activation of G_i_ proteins and inhibition of AC activity and NPR-C-mediated decrease in cAMP levels contributes to the activation of PLC signaling [130]. 

A 37-mer peptide corresponding to the cytoplasmic tail of NPR-C and a similar peptide with 10 additional N-terminal amino acids derived from receptor TM in a GTP-dependent manner inhibit AC activity in the cardiac membranes with *K*_*i*_ of about 1 nM [131]. Preincubation of the membranes with PT blocks AC inhibitory effect of peptides, which suggests that the latter, like ANP, inhibit the enzyme activity via PT-sensitive G_i/o_ proteins. To identify in the cytoplasmic domain of NPR-C the segments responsible for inhibition of AC activity the series of 8–18-mer peptides corresponding to different regions of this domain were synthesized and examined as regulators of the G_i/o_ proteins and AC activities [70, 132]. These peptides contain typical G_i/o_-activating sequences and have two basic amino acids at the N terminus and BBXB or BBXXB motifs at the C terminus [30, 133]. 

Pagano and Anand-Srivastava showed that positively charged peptides 461–472, 469–480, 469–485, and 481–492 in a concentration-dependent manner inhibit the basal AC activity with *K*_*i*_ between 0.1 and 10 nM and significantly decrease forskolin-, glucagon- and isoproterenol-stmulated AC activity in A-10 vascular smooth muscle cells and their inhibitory effects are completely attenuated by PT treatment [70] (Table 1). These peptides inhibit the stimulation of DNA synthesis induced by angiotensin II, endothelin, and arginine vasopressin but do not influence the basal level of DNA synthesis in these cells [72]. It was also shown that peptide 461–472 inhibits both the phosphorylation of ERK1/2 and AKT protein kinases and the increase of G*α*_i_ subunit expression induced by vasoactive peptide hormones. In addition, in the presence of this peptide the inhibitory effects of PD-98059, a MEK inhibitor, and of wortmannin, a PI3-K inhibitor, on hormone-induced DNA synthesis are potentiated. These data suggest that positively charged peptides containing G_i/o_-activating sequences, irrespective of their location within NPR-C cytoplasmic domain, inhibit proliferative responses of the muscle cells to vasoactive peptide hormones realized via G_i/o_ protein and MAPK/PI3-K/AKT signaling pathways [72]. 

Murthy and Makhlouf [132] synthesized and studied the related peptides 467–482, 469–485, and 479–496 corresponding to the N-terminal, middle, and C-terminal regions of the intracellular domain of NPR-C. Peptide 469–485, the same as the selective NPR-C agonist ANP(4–23), significantly increases the GTP binding to G*α*_i1_ and G*α*_i2_ subunits but not to G*α*_i3_, G*α*_s_, and G*α*_q/11_ subunits, stimulates phosphoinositide hydrolysis by activating PLC*β*3 via the G*βγ* subunits of both G_i1_ and G_i2_ proteins with an EC_50_ of 1.3 *μ*M, and inhibits forskolin-stimulated AC activity by 64% in solubilized tenia coli smooth muscle membranes. In the presence of this peptide the stimulatory effects of ANP(4–23) on G_i1_ and G_i2_ proteins binding and the PLC*β*3 activity are increased by 187, 289, and 43%, respectively. Peptide 467–482 with a partial C-terminal BBXXB-motif is a less effective activator of the G_i1_ and G_i2_ proteins and PLC*β*3. Peptide 479–496 does not influence the basal level of G protein binding and PLC activity but decreases the stimulatory effects of both ANP(4–23) and peptide 469–485 on G_i1_ and G_i2_ proteins binding and PLC*β*3 activity. Peptide 469–485 induces contraction in saponin-permeabilized smooth muscle cells and enhances the ability of ANP(4–23) to induce contraction. Peptide 479–496 has no effect on contraction but inhibits ANP(4–23)-induced contraction. It follows that peptide 479–496 inhibits G_i_ protein activation and cell responses mediated by ANP(4–23) and peptide 469–485, suggesting that the positively charged motif KHRELR^491-496^ localized at the C-terminus of this peptide mediates binding but not activation of G protein, thus, causing peptide 479–496 to act as a competitive inhibitor of G_i_ protein activation [132]. Summing up, using the synthetic peptides the selective G_i_ protein-binding and -activating determinants within cytoplasmic tail of NPR-C participating in activation of PLC-dependent signaling cascades responsible for muscle contraction were identified, which coincides very well with the later mutational and deletion studies [71].

## 5. Heterotrimeric G Proteins

Heterotrimeric G proteins consisting of *α*, *β*, and *γ* subunits are divided according to the nature of *α* subunit into four main families, G_s/olf_, G_i/o_, G_q/11_, and G_12/13_, based on structural and functional similarity [134]. In mammals there are 21 G*α* subunits encoded by 16 genes, six G*β* subunits encoded by five genes, and 12 G*γ* subunits. G*β* and G*γ* subunits form a functionally active G*βγ*-heterodimeric complex; it anchors in the membrane and does not dissociate under nondenaturing conditions. G_s_ proteins activate AC and calcium channels, G_i_ proteins inhibit AC and activate potassium channels, G_o_ proteins stimulate PLC, activate potassium channels, and inactivate calcium channels, G_q/11_ proteins activate PLC, and G_12/13_ proteins stimulate Rho/Rho kinase activity [4, 5, 10]. 

**Figure 2:**
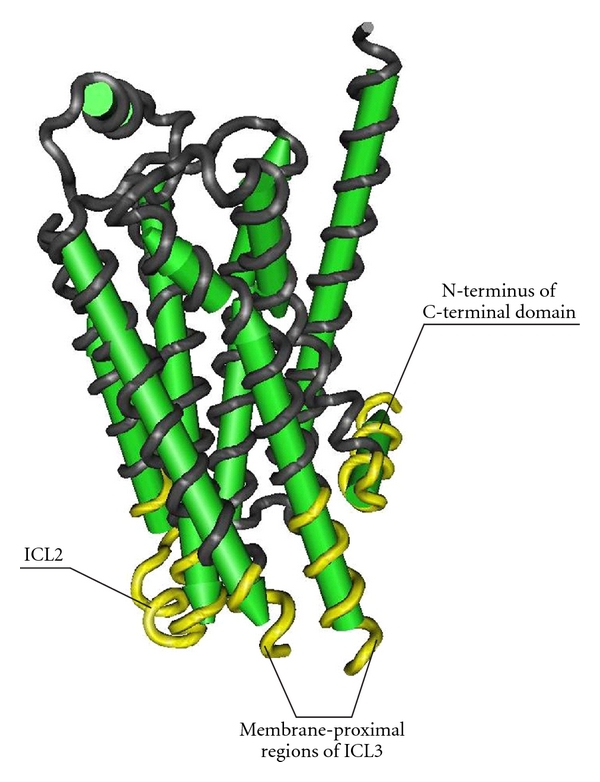
The intracellular regions of G protein-coupled receptor of the serpentine type (*yellow*) participating in the interaction with heterotrimeric G proteins.

Several different regions of G*α* subunit are implicated in recognition of GPCRs and are responsible for the specificity of receptor-G protein interaction, but the extreme C-terminal region is of special importance for effective and selective interaction of different types of G proteins with ligand-activated GPCR [7, 42, 92, 94]. This negatively charged region of G*α* binds with positively charged intracellular regions of GPCRs. It seems quite likely that GPCRs use C-terminal region as a “latch” to alter the conformation of the *α*5 helix of G*α* subunit (Figure 2). The conformational changes of the *α*5 helix give rise to a change in the conformation of the *β*6/*α*5 loop, destabilize its contacts with the guanine ring of GDP, and lead to the GDP/GTP exchange [135]. In addition to the extreme C-terminus of G*α*, the *α*4 helix, and the *α*4/*β*6 loop, both localized close to the C-terminus, also directly participate in a receptor-G protein coupling and in the case of G*α*_i_ subunit determine its specificity [32]. It was shown that an anionic cluster of G*α*_i_ subunit *α*4 helix, especially negatively charged Glu^308^ and uncharged Gln^304^, directly interact with the positively charged region in ICL-3 of G_i_-coupled D_2_-DR, and 5-HT_1A_R [32, 94]. The replacement of the residues in G*α*_i1_ subunit by the corresponding residues in G*α*_s_ (Q^304^R and E^208^L) leads to uncoupling mutant G*α*_i1_ to D_2_-DR, whereas the replacement of *α*4 helix residues in G*α*_s_ by the corresponding residues in G*α*_i1_ (R^342^Q and L^346^E) induces, on the contrary, activation of mutant G*α*_s_ by D_2_-DR. It was shown also that peptides derived from N-ICL-3 of G_i_-coupled D_2_-DR, 5-HT_1A_R and *α*_2B_-AR give a 1.5–2.5-fold increase of GTP binding of the wild type of G*α*_i1_ but have no effect on G*α*_i1_ with paired mutation Q^304^R/E^208^L. At the same time, having no influence on the GDP/GTP exchange of the wild type of G*α*_s_ they significantly increase GTP binding of the G*α*_s_ with mutation R^342^Q/L^346^E [32]. The fact that all G*α*_i/o_, unlike the other types of G*α* subunits, have an anionic cluster in the *α*4 helices gives grounds to suggest that positively charged intracellular regions of G_i/o_-coupled GPCRs interact with two anionic regions of G*α*_i/o_, the C-terminus and the *α*4 helix, while the same regions of G_s_- and G_q/11_-coupled GPCRs only with the C-terminus. Thus, the extreme C-terminus, the *α*4 helix and the *α*4/*β*6 loop of G*α* subunit are responsible for its selective interaction with receptor and account for the ability of synthetic peptides corresponding to these regions to influence signal transduction via the cognate G*α* subunit. 

Among the peptides derived from C-terminal region of the G*α* subunits the best studied is the peptide 340–350 of transducin G*α*_t_ subunit [135, 136] (Table 1). This peptide binds with photoactivated rhodopsin, meta-II-rhodopsin, and stabilizes the active signaling meta-II-rhodopsin conformation, thus mimicking the effects of transducin [73, 75]. Peptide 340–350 also interacts with meta-Ib-rhodopsin, another intermediate of light-activated receptor, forming a complex with inactive GDP-bound transducin, and with active G-protein-binding state of opsin, the ligand-free form of rhodopsin, stabilized at low pH [74, 76]. 

The peptide interacts with the inner segments of TM5, TM6, TM7, and the N-terminus of the additional helix 8 of active forms of opsin [137–139]. The interaction between peptide 340–350 with Lys^341^ replaced by leucine and the inner surface of TMC of rhodopsin leads to destruction of a hydrogen bond network which includes the side chains of Arg^135^ and Glu^134^ from the conserved E(D)RY motif localized in TM3/ICL2 interface and the side chains of Glu^247^ and Thr^251^ localized in cytoplasmic end of TM6. These events provoke the outward movement of TM6 and the formation of TM5–TM6 pair is stabilized by new interactions between the side chains of Glu^247^ and Thr^251^ of the TM6 that are released from Arg^135^ and the side chain of Lys^231^ of the TM5. The peptide binding to rhodopsin is supposed to be rate-limited by the outward movement of TM6 [135]. The contacts with inner surface of the TM5 and TM6 induce a *α*-helical conformation in peptide 340–350(K^341^L) with a C-terminal reverse turn. The carbonyl groups in the reverse turn constitute the centre of a hydrogen-bonded network, which links the two receptor regions containing the E(D)RY motif and the conserved NPxxY(x)_5,6_F motif connecting the TM7 and the helix 8. 

The analogs of C-terminal peptide 340–350(K^341^L) can stabilize meta-II-rhodopsin when they are covalently bound to one of the two native reactive cysteine residues, Cys^140^ and Cys^316^, localized on the intracellular surface of rhodopsin. Using synthetic peptides that differ in localization of a chemical crosslinking group, either at the N-terminus or the C-terminus of peptide 340–350(K^341^L), it was shown that the N-terminus of peptide analog M-23S is crosslinked to Cys^140^ and the C-terminus of peptide analog B23S-IA to Cys^316^ [75]. The peptide M-23S crosslinking to the Cys^140^ localized on the cytoplasmic tip of TM3 and the peptide B23S-IA crosslinking to Cys^316^ localized in N-terminus of helix 8 are both necessary for stabilization of meta-II-rhodopsin. At the same time, peptide B23S-IA stabilizes the more compact meta-IIa-rhodopsin whereas peptide M-23S stabilizes meta-IIb-rhodopsin, protonation form of meta-II-rhodopsin capable of activating the G protein [75]. 

Using IR-spectroscopy it was shown that negatively charged residues in the peptide 340–350 have a very important role in its interaction with rhodopsin [140]. Free carboxyl groups of Glu^342^ and Asp^346^ residues in G*α*_t_ C-terminal peptide subunit participate in specific binding with positively charged intracellular regions of rhodopsin. The elimination of the N-terminal positively charged amino group by N-acylation of peptide 340–350 and delocalization of its positive charge replacing lysines by arginines increase the affinity of modified peptide for meta-II-rhodopsin, whereas amidation of the C-terminal negatively charged carboxyl group, on the contrary, reduces it [141]. The substitution of positively charged lysine by uncharged leucine greatly increases the affinity of peptide 340–350(K^341^L), designated as 23S, for meta-II-rhodopsin and stabilizes its conformation [142]. Highly conservative in G*α*_t_ and G*α*_i/o_ subunits Cys^347^ residue, the target for ADP-ribosylation by PT, is involved in a hydrophobic interaction between peptide and meta-II-rhodopsin, since with the substitution of this cysteine by amino butyric acid, where the methylene-sulfhydryl group is replaced by an ethyl group the activity of the C-terminal peptide is maintained, whereas substitution by alanine gives a 3-fold reduction in the meta-II-rhodopsin stabilizing activity of the peptide. Alongside with this, the modification of cysteine SH-group by ribosyl radical brings about changes in the conformation of C-terminal region in full-size G*α*_t_ subunit as well as of peptide 340–350, which disturbs their interaction with rhodopsin. 

It was shown that peptides, derivatives of C-terminal sites of the G*α*_s_, very different in length disturb by the competitive mechanism the functional interaction of receptors and G_s_ proteins and change the affinity of receptors for hormones [66, 77, 143–151]. The expression of 83-mer polypeptide derived from C-terminal region of the G*α*_s_ induces the inhibition of *β*_2_-AR- and D_1A_-DR-mediated cAMP production in HEK293 cells [146]. Short synthetic peptides corresponding to progressively longer segments of the G*α*_s_ C-terminus, 384–394, 382–394, 380–394, 378–394(C^379^A), 376–394(C^379^A), and 374–394(C^379^A), stimulate specific binding of selective agonist CGS21680 to the G_s_-coupled A_2A_-adenosine receptor in the rat striatal membranes both in the presence and in the absence of GTP*γ*S, a nonhydrolysable GTP analog, which uncouples G proteins from receptors (Table 1). The most effective peptides are 378–394(C^379^A), 376–394(C^379^A), and 374–394(C^379^A), and the shortest peptide 384–394 is less active [77]. In accordance with the results of the study of G*α*_s_ crystal structure of the G*α*_s_, the *α*5-helix responsible for effective interaction with the receptors involves the region from Asp^368^ to Leu^394^ [152]. Thus, the peptide containing 17 and more amino acid residues have a stronger propensity to assume an *α*-helical conformation compared with the shortest peptides. The NMR analysis of 11-mer 384–394 and 21-mer 374–394(C379A) peptides was made and showed that both peptides demonstrate a marked propensity to form *α*-helical structure in hexafluoroacetone/water, a mixture with structure stabilizing properties, peptide 384–394 having the shortest *α*-helix between Arg^389^ and Leu^394^, and peptide 374–394(C^379^A), the longest *α*-helix spanning region from Asp^381^ to Leu^394^ [77, 153]. The addition of the most active 21-mer 374–394(C^379^A) peptide causes an increase of the number of binding sites but does not stabilize the high-affinity state of the A_2A_-adenosine receptor. Peptide 374–394(C^379^A) inhibits agonist-stimulated AC activity by 35% and does not have a significant effect on the basal and forskolin-stimulated activities. This is the evidence that the peptide disrupts the signal transduction on the stage of coupling between agonist-activated A_2A_-adenosine receptor and G_s_ protein and does not interact directly with catalytic unit of AC [77]. 

A 37-mer peptidic constrain (A42) containing a 16-mer membrane-permeable sequence (MPS) of penetratin, derived from the homeodomain of the *Drosophila melanogaster* transcription factor Antennapedia that translocates through membranes, and a 21-mer peptide 374–394(C^379^A) was designed and shown to have no affect on cell viability but to significantly inhibit adenosine receptor-mediated cAMP accumulation in PC12 cells with an EC_50_ of 5 *μ*M and decrease A_2A_-adenosine receptor- and *β*-AR-mediated cAMP production in human cell line HMEC-1 [151]. The maximal efficacy of NECA, a selective agonist of A_2A_-adenosine receptor, is substantially reduced in the presence of A42 peptide suggesting that it competes with G*α*_s_ for the interaction with receptor. The A42 peptide does not directly modulate AC activity and does not affect G_i_- and G_q_-coupled receptor signaling, which indicates that the specific target for peptide is the receptor G_s_-binding and -activating surface. Using NMR analysis it was shown that A42 peptide is arranged in two stretches of *α*-helical structure encompassing sequences 3–10 and 18–36. Even in a membrane-mimicking environment and in conjugation with MPS, the segment 18–36 has the secondary structure resembling those of peptide 374–394(C^379^A) and of C-terminal region in full-size G*α*_s_ [151]. The helix 18–36 is amphipathic and has a polar surface lined by Asp^378^, Arg^390^, Asp^381^, and Arg^385^ and two hydrophobic surfaces formed by less polar residues Ile^382^, Ile^383^, Met^386^, His^387^, Tyr^391^, and Leu^393^. The N-terminal penetratin fragment of A42 peptide also possessing *α*-helical structure has hydrophobic and hydrophilic residues forming clustered surfaces at the N-terminus and in the center of the fragment and is responsible for peptide internalization by endocytosis. 

The peptide strategy can be used to study the functional role of distinct amino acid residues in the C-terminus of G*α*_s_. The leucine residue localized at position (−2) is absolutely invariant in C-terminal regions of all G*α* subunits. The substitution of this leucine (Leu^393^) with Ala, Phe, Lys, and D-Leu in C-terminal peptide 384–394 from the G*α*_s_ C-terminus gives modified peptides possessing much lower activity compared with unmodified peptide [147]. However, the peptides in which Leu^393^ was replaced by Asp and Thr retain a residual activity. The conformational analysis of peptides indicates that the side chain of Leu^393^, together with side chains of Ile^382^ and Met^386^, is involved in formation of a hydrophobic surface that is very important for effective interaction with the cognate receptor. 

Minigene plasmids encoding 11-mer peptides corresponding to the C-terminal regions of G*α*_i_, G*α*_o_, G*α*_q_, G*α*_12_, and G*α*_13_ have been successfully used to discern the contribution of different types of G proteins to signal transduction via m_2_-MChR and thrombin receptors [154–156]. Plasmid minigene vectors encoding the C-terminal sequence of G*α* serve as competitive inhibitors presumably by blocking the G protein-binding site on the GPCR that normally binds the G protein. A minigene vector expressing the G*α*_i1/2_ C-terminal peptide specifically inhibits G_i1/2_ protein inwardly rectifying K^+^ channel activity following agonist stimulation of the m_2_-MChR and decreases thrombin-induced inhibition of AC activity in endothelial cells [154, 155]. The minigene vectors expressing the G*α*_o_ and G*α*_q_ C-terminal peptides in a competitive manner block the coupling between G_o_ and G_q_ proteins and the human thromboxane A_2_ receptor (TXA_2_R); they significantly decrease thrombin-stimulated intracellular Ca^2+^ level and inhibit the stimulatory effects of thrombin on PLC activity; as a result PI 1,4,5-P_3_ production is blocked [155, 156]. The minigenes encoding the C-terminal peptides of G_12_ and G_13_ proteins decrease thrombin-stimulated stress fiber formation that is mediated via G_12/13_ protein family [155]. A minigene encoding mutated C-terminal sequence of G_q_ protein, in which two C-terminal residues, Ala and Val, are replaced by the Thr and Lys, has a little effect on thrombin-induced PLC activity, and even partially restores the response of the enzyme decreased in the presence of wild G*α*_q_ C-terminal peptide [155]. A minigene expressing 15-mer peptide of G*α*_q_ C-terminal region, the same as 11-mer peptide, inhibits thrombin-stimulated PLC activity, and, in addition, specifically binds to ligand-free TXA_2_R with high affinity (*K*_*d*_ is 17 *μ*M) and to agonist-activated receptor with low affinity (*K*_*d*_ is 240 *μ*M) [157]. It is a clear indication that the C-terminal region of G*α*_q_ binds to TXA_2_R without agonist, which suggests the existence of a preoccupied G protein-binding site on the intracellular domains of ligand-free TXA_2_R. The action of minigenes is very specific, being realized that provided hormonal responses are mediated via their cognate G proteins. The above is the evidence that the use of minigene-based peptide strategy not only confirms the view that each G protein can control certain signaling events but also emphasizes, in addition, the specificity of the GPCR-G protein interface. Thus, the minigenes encoding G*α* C-terminal regions appear to be a powerful tool for dissecting out the G protein that mediates a given physiological function following thrombin activation. 

As mentioned above for G*α*_s_-derived A42 peptidic constrain containing the MPS, the efficiency of G*α* C-terminal peptides depends on the ability of the latter to penetrate the plasma membrane. A shorter peptide 350–359 corresponding to the extreme C terminus of G*α*_q_ modified by MPS corresponding to Kaposi fibroblast growth factor signal region inhibits 5-HT_2C_R-mediated signaling in cultured choroids plexus epithelial cells significantly decreasing serotonin-stimulated PLC activity, whereas its unmodified membrane-impermeable analog is unable to disrupt the functional coupling between agonist-activated 5-HT_2C_R and G_q_ protein [145]. A peptide 385–394, derivative of the C terminus of G*α*_s_, modified by this MPS at the N-terminus significantly decreases, to almost basal level, *β*_2_-AR agonist-induced stimulation of AC activity in astrocytes, whereas the nonconjugated analog that is unable to penetrate the plasma membrane has low inhibitory activity. It should be pointed out that Kaposi fibroblast growth factor-derived MPS alone is inactive; therefore its contribution in the blockade of any signal cascade tested is hardly of great importance. However, it is not always the case. We showed that several polycationic peptides used as MPSs can have influence on the activity of the components of hormonal signaling systems, G proteins and AC in particular, and their action must be taken into account in developing cell-penetrating conjugates on the basis of these peptides [51, 158–160]. 

The interaction of G proteins with the effector enzymes, AC in particular, is also very important for deciphering the molecular mechanisms of GPCR-mediated signal transduction. Proceeding from the data that the crystal structure of G*α*_s_ complexed with the soluble functional fragments of AC shows three regions localized in G*α*_s_, all having a direct contact with the enzyme [161], three peptides 199–216, 222–247, and 268–286 corresponding to these regions were synthesized and their influence on the activity of the types 2 and 6 ACs studied [78] (Table 1). An 18-mer peptide 199–216 encoding the Switch I region from G*α*_s_ stimulates the basal and forskolin-stimulated activities of AC2 as well as AC6, and forskolin does not appear to shift the peptide concentration-effect curves. This peptide also inhibits the G*α*_s_-stimulated activity of both isoforms of the enzymes by about 30% and, thus, behaves as a partial agonist. At the same time, the analogous peptide with the substitutions G^206^P, I^207^D, E^209^K, and K^211^A has no effect on AC2 and AC6 activities. The residue Ile^207^ of G*α*_s_ makes a contact with AC while the residues Gly^206^, Glu^209^, and Lys^211^ are responsible for appropriate local conformation of the Switch I region. Peptide 222–247 encoding the Switch II region, the same as peptide 199–216, stimulates the basal activity of both AC isoforms making it 2-3 times higher, significantly increases forskolin-stimulated AC activity, and inhibits G*α*_s_-stimulated AC activity by 30–35%. Peptides 199–224 and 204–229 encoding the Switch II regions of the G*α*_i_ and G*α*_q_, respectively, that structurally are very similar to the Switch II region of G*α*_s_ are not capable of stimulating the AC activity [78]. The effects of peptides 199–216 and 222–247 on the activity of AC2 are additive at lower concentrations giving a left-shifted curve but are not additive at saturating concentrations. A different effect is observed for AC6 where peptide 222–247 lowers the effect of peptide 199–224. In the case of AC2 the stimulating effect of peptide 199–224 is stronger compared with that of peptide 222–247 while in the case of AC6 the effect of peptide 222–247 predominates. Now a suggestion can be put forward that the molecular mechanisms underlying interaction of the Switch regions of G*α*_s_ and AC isoforms are not all alike. Peptide 268–286 corresponding to *α*3-*β*5 region that interacts with the C-terminal tail as well as with the central cytoplasmic loop of AC inhibits the basal and forskolin-stimulated activities of both enzymes by about 30% and, in addition, significantly decreases the G*α*_s_-stimulated AC activity. Potent inhibition of the G*α*_s_-stimulated activity by peptide 268–286 indicates that *α*3-*β*5 is involved in stabilization of the appropriate orientation of the Switch regions required for effective AC activation. The peptide with the highly conservative residues Trp^277^ and Trp^280^ replaced by Arg and Lys, respectively, has no influence on AC activity [78]. Studying the G*α*_s_-derived peptides, specific functions of different regions of G*α*_s_ directly contacting AC were revealed.

## 6. Adenylyl Cyclases

Mammalian membrane-bound ACs producing the second messenger cAMP in response to G_s_ protein stimulation are represented by at least nine different isoforms (AC1–AC9) and possess two hydrophobic transmembrane regions, each built up by six TM and two pseudosymmetric cytosolic domains C_1_ and C_2_ that comprise subdomains C_a_ and C_b_. All AC subtypes are stimulated by GTP-bound G*α*_s_ and diterpene forskolin, AC1 by Ca^2+^-calmodulin (CaM), AC2 by G*βγ*-dimer, while AC5, and AC6 are inhibited by submicromolar concentrations of Ca^2+^ and AC1, AC5 and AC6 by GTP-bound G*α*_i_ subunit. Synthetic peptides are widely used for identification of the regions in AC cytoplasmic domains involved in the functional interaction with the regulatory proteins and responsible for the enzyme activity [79–81] (Table 1). 

To determine the role of various interaction regions within the C_1_ and C_2_ domains of the AC2 and AC6 in the basal and G*α*_s_-, forskolin- and Mn^2+^-stimulated activities, five peptides corresponding to different interface regions involved in interaction with each other were synthesized [81]. Two peptides, 427–444 and 487–511, correspond to *α*2-*β*2-*β*3 and *β*4-*β*5-*α*4 regions of the C_1_ domain of AC6, and three peptides, 899–926, 927–948, and 984–1015, to *α*2, *β*2-*β*3, and *α*3-*β*4 regions of the C_2_ domain of AC2, respectively. All these peptides inhibit both G*α*_s_- and forskolin-stimulated activities of full-length AC2 and AC6 expressed in Hi5 cells. Peptides 487–511 and 984–1015 also inhibit basal activity by 30%. Peptide 899–926 encoding C_2_ domain *α*2 region inhibits the basal and Mn^2+^-stimulated activities of AC2 and AC6 by 52–59%, and G*α*_s_- and forskolin-stimulated activities of the enzymes by 55–63 and 71–80%, respectively. The other peptides do not affect Mn^2+^-stimulated activities significantly. The peptides with the replacement of residues most important in the interaction with other amino acids in the C_1_ and C_2_ domains, such as 427–444(R^434^D, K^436^D, I^437^P), 487–511(G^498^P, K^501^E, Q^503^R), 899–926(E^910^K, R^913^P, N^916^A, E^917^K, L^926^S), 927–948(K^936^D, I^937^F, K^938^D, T^939^F, I^940^F), and 984–1015(H^989^D, F^991^K, N^992^K, K^995^G, I^1010^D, G^1011^D, A^1012^Q, Q^1013^K) have no influence on the enzyme activity. Thus, the peptide strategy suggests that the AC activity stimulated by different regulators involves distinct interface interactions in the full-length enzyme, which indicates that the AC may have multiple catalytically competent configurations [81]. 

To identify CaM-binding regions in CaM-regulated AC1 and to decipher the molecular mechanisms of CaM regulatory action on the enzyme activity, the peptides corresponding to C_1b_ subdomain region 495–522 (pAC28) of AC1 and C_2a_ subdomain region 1024–1044 (pVLG) of AC1 were synthesized [79, 80]. The peptide pAC28 exhibits a high affinity for CaM binding (*K*_*d*_, 2 nM) and inhibits CaM-stimulated AC activity with IC_50_ equal to 500 nM [79]. The peptide pVLG binds CaM with low affinity and inhibits CaM-stimulated AC activity with IC_50_ equal to 10 *μ*M, which points to a minor role of the region 1024–1044 in CaM-mediated stimulation of AC1 activity compared with the region 495–522. However, despite its rather high affinity for CaM, the peptide pAC28 binds to CaM in a Ca^2+^-independent manner, although the modulation of AC1 activity by CaM is known to be very Ca^2+^-dependent. At the same time, the peptide pVLG binds to CaM in a Ca^2+^-dependent manner. These data speak in favor of the fact that CaM requires not only Ca^2+^-independent C_1b_ subdomain region 495–522 but also Ca^2+^-dependent C_2a_ subdomain region 1024–1044 to stimulate AC1. When two CaM-critical residues of the peptide pVLG, Val^1027^ and Leu^1030^, are replaced by alanines, the specific binding of modified peptide pAAG with CaM is significantly reduced and the peptide pAAG competes with AC for CaM less efficiently than the peptide pVLG, pointing to a sequence dependence of the region 1024–1044 for binding to CaM. Thus, despite the structural and functional variety and different recognizing motifs on AC, CaM, like G*α*_s_ and G*βγ* subunits, and forskolin, modulates the enzyme activity according to a two-site-interaction mechanism [80]. The peptide pVLG, in addition to inhibiting CaM-stimulated AC activity, also decreases the basal activity of AC1. The peptide pAAG having the inactive amino acid substitutions is also able to inhibit the enzyme activity. This is likely to be due to the interaction of peptide 1024–1044 with complementary region forming catalytic and/or regulatory sites of AC1 in CaM-free state.

## 7. The Family of Phospholipases C

Inositol-specific PLCs are the family of Ca^2+^-dependent multidomain enzymes, represented in mammalian cells by several isoforms, such as PLC*β*, PLC*γ*, PLC*δ*, PLC*ε*, PLC*ζ*, and PLC*η*. They catalyze the hydrolysis of phosphatidylinositol 4,5-diphosphate (PI 4,5-P_2_) to yield diacylglycerol and phosphatidylinositol 1,4,5-triphosphate (PI 1,4,5-P_3_), which are important secondary messengers. PI 1,4,5-P_3_ binds to its receptor in the endoplasmic reticulum to open Ca^2+^ channels which increases the intracellular level of Ca^2+^ and activates calcium-sensitive enzymes, such as protein kinase C (PKC) [162, 163]. The PLC activity is regulated by many hormones and growth factors that realize their effects through GPCRs, receptor tyrosine kinases, and small G-proteins of the Ras and Rho families. These enzymes are implicated in intracellular signal transduction, vesicle transport, endocytosis, exocytosis, regulation of the ionic channels functions, mitosis, and cytoskeletal reorganization. 

The family of PLC*β*, the major effectors of G_q_ proteins, consists of four members different in their response to G proteins. The PLC*β*1, for instance, is activated by GTP-bound G*α*_q_ subunit and is insensitive to G*βγ* dimer, and the activity of PLC*β*2, on the contrary, is regulated by G*βγ* dimer. The PLC*β*1 is expressed as two splice variants, PLC*β*1a and PLC*β*1b, differing only in the extreme C-terminal sequences, 75 amino acid residues in PLC*β*1a and 32 in PLC*β*1b. The PLC*β*1a is localized in the cytoplasm, whereas PLC*β*1b is associated with the sarcolemmal membrane, but deletion of the entire unique 31-mer sequence causes cytosolic localization of mutant PLC*β*1b. Using synthetic peptides it was found that the critical role in association with the membrane has a short 10-mer C-terminal segment of PLC*β*1b that determines functional activity of the enzyme [82] (Table 1). A myristoylated peptide corresponding to this segment of PLC*β*1b enriched by proline residues induces the dissociation of the enzyme from the sarcolemmal membrane and inhibits its response to *α*_1_-AR agonists with EC_50_ of 12 *μ*M, whereas the same peptide from the C-terminus of PLC*β*1a taken as control is ineffective. Later it was shown that a 32-mer C-terminal peptide of PLC*β*1b competes with full-size enzyme for sarcolemmal localization and inhibits PLC activity stimulated by bsoth *α*_1_-AR agonist norepinephrine and expressing constitutively active G*α*_q_ [83]. A 32-mer PLC*β*1b-peptide significantly decreases a substantial hypertrophic response induced by phenylephrine and mutant G*α*_q_ subunit. So, PLC*β*1b-peptides preventing the enzyme association with the sarcolemma and inhibiting signal transduction via G_q_-coupled receptors downstream of G_q_ activation may provide useful therapeutic agents for the treatment of hypertrophy and other cardiomyocyte damages. The inhibition of hypertrophic signal transduction may be also achieved using the peptides, derivatives of G*α*_q_, that are able to prevent pressure overload hypertrophy and to delay the progression of heart failure [164, 165]. However, the inhibition of G_q_-coupled signaling pathways involved in control of fundamental cellular processes at the level of G_q_ protein far from being an ideal strategy because it can induce numerous dysfunctions in these pathways and, therefore, the selective inhibition or stimulation of GPCR or the enzyme, the generators of second messengers, PLC*β*1 in particular, is preferable in this case. 

The PLC*β*-peptides are used for identification of the G protein-interacting regions in PLC*β* [145, 166]. The PLC*β*1-peptide 1053–1084 modified at the N-terminus by MPS corresponding to Kaposi fibroblast growth factor signal region in a dose-dependent manner inhibits GTP*γ*S-dependent activation of PLC*β*1 in both purified PLC*β*1 and crude membrane preparations containing the intact enzyme, which indicates the disruption of the functional interaction between PLC*β*1 and G*α*_q_ in active, GTP-bound, form [145]. Peptides 564–583 and 574–593 corresponding to the central region of PLC*β*2 specifically bind to G*βγ* and inhibit G*βγ*-mediated stimulation of the enzyme activity [166]. Moreover, peptide 564–583 is more active compared with peptide 574–593, which speaks in favor of the fact that region 564–583 contains all determinants required for effective G*βγ* dimer binding. The modification of peptide 564–583 with MPS does not influence their binding properties but increases its ability to block G*βγ*-mediated activation of the enzyme activity in cultured cells [145]. 

The PLC*β*-peptides are appropriate tools for identification of the enzyme isoforms participating in signal transduction triggered by different hormones that activate G_q_- and G_i/o_-coupled GPCRs [84, 145]. The PLC*β*1-peptide 1053–1084 modified by MPS blocks, down to the basal level, serotonin-induced phosphatidylinositol hydrolysis in cultured choroids plexus epithelial cells, where G_q_-coupled 5-HT_2C_R is the main mediator of serotonin stimulation. The inhibitory effect of peptide MPS-1053–1084 is dose-dependent with IC_50_ equal to 55 *μ*M. The PLC*β*2-peptide MPS-564–583 does not significantly influence 5-HT_2C_R-mediated stimulation of phosphatidylinositol hydrolysis. These data indicate that serotonin-induced activation of the 5-HT_2C_R leads to the release of active G*α*_q_ subunit and its interaction with PLC*β*1 specifically regulated by G*α*_q_, but not with PLC*β*2 regulated by G*βγ* dimer [145]. The same happens in the case of metabotropic glutamate receptors (mGluRs). At concentration 300 *μ*M peptide MPS-1053–1084 significantly inhibits phosphatidylinositol hydrolysis stimulated by 3,5-dihydrophenylglycine, a specific agonist of the types 1 and 5 mGluR (mGluR1 and mGluR5), in primary cultures of astrocytes where G_q_-coupled mGluR5 is expressed. In contrast, the treatment of the cells with peptide MPS-564–583 does not decrease the stimulation of mGluR-mediated phosphatidylinositol hydrolysis. These data suggest that mGluR5-mediated signaling is involved in PLC*β*1 activated by G*α*_q_ [145]. 

Microinjection of heptapeptide 1161–1167 corresponding to the extreme C-terminal region of PLC*β*3 in the cytoplasm of the HN33 hippocampal cells leads to a significant decrease of calcium signaling induced by mGluR agonist N-1,3-cyclopentanedicarboxylic acid [84] (Table 1). Injection with the same C-terminal peptides of other PLC*β* isozymes, such as PLC*β*1, PLC*β*2, and PLC*β*4, does not cause any changes in the calcium signaling. As according to the available data, the C-terminus of the PLC*β*3 binds to adaptor and regulatory proteins, a PDZ domain-containing multimodular scaffolding protein Shank/ProSAP in particular, mediating the interaction between ligand-activated mGluR, G_q_ protein, and PLC*β*3 at highly specialized submembranous sites, it can be assumed that peptide 1161–1167 competes with the enzyme for binding with these proteins and, thus, inhibits the transduction of a hormonal signal from the receptor to the enzyme [84]. 

The membrane-permeable PLC*β*-peptides, specifically binding G*α*_q_ and G*βγ*, can be used to study the involvement of these subunits in PLC-independent signaling pathways [145]. Pretreatment of HEK cells expressing the G_i/o_-coupled *α*_2A_-AR with PLC*β*2-peptide MPS-564–583 that specifically binds G*βγ* dimer completely blocks the activation of MAPK by epinephrine through *α*_2A_-AR, while the PLC*β*1-peptide MPS-1053–1084 specifically interacting with G*α*_q_ does not affect *α*_2A_-AR-mediated MAPK activation. Unlike this, pretreatment of HEK cells expressing the G_q_-coupled thrombin receptor with MPS-1053–1084 blocks the subsequent activation of PLC*β* by thrombin receptor-activating peptide, but peptide MPS-564–583 in this case is ineffective. These data show that activation of MAPK cascade by *α*_2A_-AR agonists is mediated through G*βγ* subunits released from the G_i/o_ proteins, while the PLC*β* activation by thrombin receptor agonists involves G*α*_q_, but not G*βγ*, which coincides with the data obtained using the other methods [145]. 

Studying synthetic peptides corresponding to different loci of PLC*δ*1, putative CaM-binding motif 473–493 of IQ type (VQ*XXX*K*XXXX*K) was identified in the X/Y linker region of the enzyme that was not detected in the other PLC isoforms [86] (Table 1). Peptide 473–493 in a competition manner inhibits the binding of CaM with PLC*δ*1, but only in two cases, either in the absence of or at low concentrations of Ca^2+^. The binding of CaM to peptide 473–493 is, indeed, inversely related to Ca^2+^ concentration, while the peptide coprecipitates PLC*δ*1 irrespective of Ca^2+^ concentration. The incubation of PLC*δ*1 with 10–100 *μ*M of the peptide causes a 22–53% reduction in the ability of PLC*δ*1 to catalyze hydrolysis of PI 4,5-P_2_. The results obtained with peptide 473–493 show that CaM binds with putative IQ motif located within the catalytic site of PLC*δ*1 and inhibits the enzyme activity [86]. 

In Sertoli cells FSH induces an immediate Ca^2+^ influx via a G_s_/AC/cAMP-independent signaling pathway involving PLC*δ*1 and tissue transglutaminase possessing GTP-binding and GTPase activities and designated as G*α*_h_ [167]. The myristoylated peptide corresponding to region 747–763 of PLC*δ*1 in a competition manner inhibits the enzyme activity acting on the stage of coupling between FSH-stimulated G*α*_h_ protein and PLC*δ*1, while U73122, a specific nonpeptidic inhibitor of PLC, directly interacts with the enzyme and blocks its translocation from cytosol to the plasma membrane, which is necessary for PI 1,4,5-P_3_ production (Table 1). The pretreatment of rat Sertoli cells with 0.1 and 1 *μ*M of the peptide leads to a 50 and 90% reduction of FSH-induced intracellular PI 1,4,5-P_3_ generation and to a 35 and 70% reduction of the FSH-evoked Ca^2+^ influx, which demonstrates a critical role of G*α*_h_/PLC*δ*1/PI 1,4,5-P_3_ pathway in the FSH-induced Ca^2+^ influx in Sertoli cells [87, 167]. The action of the peptide is pathway-specific, since it does not interfere with FSH-induced activation of G_s_/AC signaling pathway leading to an increase of the intracellular cAMP level. 

The PLC*γ* participates in the signal cascades involving tyrosine kinase receptors, that is, brain-derived neurotrophic factor receptors and nerve growth factor receptors, and nonreceptor tyrosine kinases, for example, B-cell receptors and T-cell receptors, as the sensor components, and scaffolding molecules, Bruton's tyrosine kinase in particular, that mediate the functional coupling between ligand-activated receptor proteins and PLC*γ* [168]. As is known, the PLC*γ* contains adjacent to its SH2 and SH3 domains a PLC inhibitor (PCI) region, including a positively charged segment YRKMRLRY, that strongly suppresses PI 4,5-P_2_ hydrolyzing activity. Synthetic peptides identical to the PCI region inhibit the enzyme activity induced not only by PLC*γ*, but also by PLC*β* and PLC-*δ* [169]. In addition, they inhibit Ca^2+^-dependent PLC activation in digitonin-permeabilized cells and agonist- and GTP*γ*S-dependent PLC activation in purified plasma membranes [170]. The modification of PCI-peptide 724–736 by the conjugating myristic acid at the N-terminus increases its PLC inhibitory effects *in vitro* [85] (Table 1). The *K*_*i*_ values are 120 and 3.5 *μ*M for unmodified peptide 724–736 and its myristoylated analog, respectively. Myristoylated peptide 724–736 at concentrations in the sub-micromolar range significantly suppresses *in vitro* PI 1,4,5-P_3_ formation induced by hormones and growth factors (EGF, PDGF, bombesin) in Swiss 3T3 fibroblasts (the IC_50_ values are rather low, approximately 1 *μ*M) and strongly inhibits cell proliferation induced by these stimuli *in vivo* in *erbB*-, *v-raf*- and *B-raf*-transformed NIH 3T3 cells, neutrophils, isolated pancreatic cells, and Swiss 3T3 fibroblasts. This indicates that myristoylation of PCI-peptides leads to structural alteration, resulting in their effective entry into substrate-containing vesicles and close association with PLC. The inhibitory effects on the basal PLC activity and hormone-stimulated PI 1,4,5-P_3_ formation and proliferation of N-myristoylated peptide 724–736 with the replacement of the Tyr^726^ and Tyr^734^ by phenylalanines are much less potent than the effects of the original myristoylated peptide. This gives grounds to say that biological activity of PCI-peptides depends to a large extent on tyrosine residues [85]. There is one thing to be mentioned at this point that unmodified peptide 724–736 with the Y726F/Y734F replacement is not active at all.

## 8. Phosphatidylinositol 3-Kinase

Heterodimeric PI3-K consisting of regulatory p85 and catalytic p110 subunits generates phosphoinositide second messengers, such as phosphatidylinositol 3,4,5-triphosphate (PI 3,4,5-P_3_) and phosphatidylinositol 3,4-diphosphate (PI 3,4-P_2_), and regulates an array of signaling pathways through the membrane recruitment and activation of downstream effector proteins [171]. It was shown that C-terminal SH2 domain of PI3-K p85*α* subunit contains PI 3,4,5-P_3_-binding region 18–29 and displays discriminative affinity for this phosphoinositide, depending on the length of acyl chain and the charge density on the phosphoinositol ring of PI 3,4,5-P_3_. Peptide 11–29 including the region selectively binds PI 3,4,5-P_3_ with *K*_*d*_ of 30 *μ*M, as in the case of the full-length C-terminal SH2 domain, but has the binding affinity for other phosphoinositides, PI 3,4-P_2_ in particular, which is one to two orders of magnitude lower compared with PI 3,4,5-P_3_ [88] (Table 1). This peptide in a dose-dependent manner inhibits the binding of [^3^H]di-C_8_-PI 3,4,5-P_3_ to the full-length C-terminal SH2 domain of PI3-K p85*α* subunit immobilized onto streptavidin-coated beads with IC_50_ of 62 *μ*M. On the addition of PI 3,4,5-P_3_ the contribution of *α*-helical conformation in the peptide structure increases significantly. Besides, confirming the fact that the binding site for PI 3,4,5-P_3_ is localized within region 11–29, these findings suggest that the other regions of p85 subunit are not involved in this binding [88].

## 9. Protein Kinase C

The family of the PKCs, intracellular serine/threonine protein kinases, includes classical forms of the enzymes (PKC*α*, PKC*β*, and PKC*γ*) undergoing activation in the presence of diacylglycerol and calcium ions, novel isoforms of PKC (*δ*, *ε*, *η*, *θ*) independent of Ca^2+^ regulation, and atypical PKC isoforms (*ζ*, *ι*) independent of diacylglycerol and Ca^2+^ [172]. All PKCs possess C-terminal serine/threonine protein kinase domain linked to a regulatory domain including an inhibitory region, designated as the pseudosubstrate site [173]. As is known, PKCs are inactive in the absence of activating agents owing to an intramolercular interaction between a short inhibitory sequence located in the pseudosubstrate site and the substrate-binding region of the catalytic domain. Synthetic peptides corresponding to pseudosubstrate inhibitory sequence selectively decrease functional activity of PKC [174, 175]. To penetrate into cell pseudosubstrate peptides were subjected to modification using MPS derived from Antennapedia protein or HIV-1 TAT protein and hydrophobic fatty acid radicals [89, 176, 177]. As a rule, such modification has no influence on cellular processes, neither does it change the spectrum of inhibitory properties of pseudosubstrate peptides. However, cell-permeable pseudosubstrate peptides from PKC*α*/*β*, PKC*η*, PKC*ζ*, and kinase domain of EGF receptor modified by myristoyl radical, in addition to inhibiting PKC activity, possess the ability to activate ERK1/2 and p38 MAPK, the components of MAPK cascade, and the intracellular enzymes (AKT kinase, in particular) involved in endothelial nitric oxide synthase activation and NO production [89, 178] (Table 1). Because an increase of endothelial nitric oxide synthase activity seems beneficial in the case of many diseases such as ischemic stroke, hypertension, and atherosclerosis, PKC-derived myristoylated peptides have a good chance to be used as pharmacological compounds for acute activation of the enzyme.

## 10. The Molecular Mechanisms of Action of Intracellular Signal Protein-Derived Peptides

According to the present view, there may be three molecular mechanisms of action of signal protein-derived peptides, all including different models of the competition relationships between the peptide and its cognate signal proteins. 

The first mechanism is based on the competition between the peptide and its cognate full-length signal protein for the interaction with the other functionally and structurally complementary signal and proteins, components of signaling cascade. According to this mechanism, GPCR-peptide directly interacts with G protein subunits, G*α* or G*βγ*; G*α*-peptide with the G*βγ*-dimer complex, the receptor or the enzyme generating the second messenger; AC-derived peptide with G protein or AC-competent regulatory proteins, CaM in particular; and so on. This creates conditions for GPCR-peptide corresponding to G protein-activating region of the receptor to activate G proteins in a dose-dependent manner and trigger coupled to them signaling cascades and in a competition manner to disturb functional interaction between its cognate ligand-activated receptor and G proteins, decreasing or completely blocking the signal transduction. Then GPCR-peptide will function as antagonist of GPCR-signaling. A peptide corresponding to the extreme C-terminal region of G*α* subunit binds to G protein-activating region of GPCR and selectively inhibits the transduction of hormonal signal from ligand-activated receptor to its cognate G proteins, acting also as antagonist of signal transduction. The peptide, derivative of the C-terminal region of the PLC*β*3, competes with the full-size enzyme for binding with adaptor and regulatory proteins, including scaffolding protein Shank/ProSAP, which leads to destabilization of functionally active receptor-G_q_ protein–PLC*β*3 complex and to inhibition of hormone-induced stimulation of phosphoinositide hydrolysis [84]. 

The second mechanism includes the interaction of synthetic peptide with complementary regions of protein homologous to it. According to Covic and colleagues, this mechanism accounts for the ability of pepducins to activate or, contrary to this, to inhibit signal transduction via their cognate receptor [97]. When pepducin acts as agonist, it binds to the high-affinity activating site formed by the intracellular regions of GPCR and, thus, stabilizes or induces the active state of receptor, which makes it possible for the latter to interact effectively with G proteins and trigger signaling cascade. When pepducin acts as antagonist, it binds to lower-affinity inhibiting site that blocks the interaction between ligand-activated receptor and G protein and prevents the transduction of signal to G protein. According to this two-site mechanism, GPCR-peptide specifically interacts with complementary regions of its cognate receptor that in intact receptor have contacts with the region homologous to the peptide affecting their mobility and rearrangement. The peptides corresponding to various regions within the C_1_ and C_2_ domains of the AC2 and AC6 differ in the ability to influence the basal and forskolin-, G*α*_s_- and Mn^2+^-stimulated activities, which indicates that these peptides interact with different complementary regions forming catalytic and regulatory site of the enzyme. 

The data obtained in our and the other authors' investigations show that the receptor specificity of GPCR-peptides and the dependence of their action on the receptors homologous to them unambiguously speak in favor of the second mechanism of action of GPCR-peptides [26, 39, 50, 97]. The receptor specificity and the need in cognate receptor are likely to be both responsible for participation of the cognate receptor in realization of regulatory effects of GPCR-peptides, which resembles the action of signal protein-derived peptides carried out by the second mechanism. 

The data about high receptor specificity of pepducins, the lipidated GPCR-peptides, are mentioned before. Studying 5-HTR-derived peptides we found that they affect only their cognate 5-HTR and practically do no influence the activity of structurally close to them 5-HTRs [39]. 5-HT_6_R-peptide 258–268 reduces the stimulating effects of serotonin and, to a greater extent, EMD-386088, a selective 5-HT_6_R agonist, on AC activity and G_s_ proteins GTP binding. At the same time, the agonists of the other types of 5-HTR, which are also able to stimulate AC system, still have such effects. The inhibitory effect of peptide 258–268 on serotonin signaling is blocked in the presence of 5-HT_6_R antagonists SB-271046 and methiothepin but is retained in the presence of antagonists of the other types of 5-HTR. 5-HT_1B_R-peptide 300–316 decreases AC inhibiting effects of serotonin and 5-HT_1_R agonists, especially of selective 5-HT_1B_R agonist 5-nonyloxytryptamine, and their stimulating effects on the G_i_ proteins, while the effects of agonists of the other types of 5-HTR in the presence of this peptide do not change. 5-HT_1B_R antagonists reduce or completely block the effects of peptide 300–316, whereas the antagonists of the other types of 5-HTR in this case are not effective [39]. 

As mentioned above, the action of P1pal-19 corresponding to N-ICL3 of PAR1 on PLC*β* and the other downstream effector enzymes is partially or completely blocked in cultured cells and tissues where the cognate PAR1 is absent. Peptide P1pal-19 and its analogs are ineffective in the presence of the mutant PAR1 entirely devoid of CTD. It follows that activation of the G proteins by P1pal-19 requires the presence of the CTD of the receptor and it may provide a binding site for PAR1-derived pepducins [98]. Peptide 615–629 derived from relaxin receptor RXFP1 stimulates the basal AC activity in the rat myocardium and brain with abundant RXFP1 receptors, but it does not affect the enzyme activity in the skeletal muscles [50], where relaxin regulates AC via the receptor of a different type [179]. The action of 5-HTR-peptides on AC system is well expressed in the brain, the main target of serotonin, and rather little in the muscle and reproductive tissues, where the 5-HTRs are expressed weakly or not at all [39]. 

The third mechanism of action of signal protein-derived peptides is based on their ability to influence the stability of homo- and heteromeric complexes formed by their cognate proteins, which in many cases accounts for the functional activity of these proteins. It is shown that the cyclic peptide 225–273 constructed on the basis of a full-size ICL3 of the V_2_-vasopressin receptor induces a significant inhibition of the bioluminescence resonance energy-transfer signal between constrains V_2_ receptor-luciferase and V_2_ receptor-yellow fluorescent protein, indicating that it modifies the distance and/or orientation between these constrains engaged in formation of dimeric complex and, thus, causes a dose-dependent decrease in specific binding of [Arg^8^]-vasopressin as a result of transition of the receptor high-affinity to low-affinity state and inhibits vasopressin-induced stimulation of AC activity [55]. Peptide 260–276 corresponding to C-ICL3 of D_2_-DR also affects the stability of the heterodimeric complex between D_1_- and D_2_-DR, which leads to alteration of dopamine signaling in the brain [32, 40, 42]. There is a view that peptide competes with its corresponding region in intact D_2_-DR containing a positively charged segment RRRRKR^217-222^ for binding to the anionic site located in CTD of D_1_-DR and prevents the D_1_/D_2_-heterodimerization. So, GPCR-peptides change the stability of receptor complex and are capable of acting either as positive allosteric regulators inducing the appropriate cellular response to agonist even at a very low concentration or as negative allosteric regulators blocking partially or completely this response. 

Concerning the mechanism of action of signal protein-derived peptides realized in the cell, it is likely be a combination of the above mechanisms, depending on the structure of the peptide and localization of the region homologous to it in cognate protein, the modification of peptides by hydrophobic radicals and functional groups, the ability of signal proteins to form intermolecular complex, and other things. Finally, the choice between the first and the second mechanisms has a direct relation to the affinity of peptide for the proteins homologous to it or the proteins-partners functionally interacting with the latter. If the affinity of synthetic peptide for complementary regions of the cognate protein is higher than that for those of other proteins, the preference is given to the second mechanism; if the affinity for complementary regions of other proteins specifically interacting with the cognate protein is above that for the latter, the first mechanism comes into action, as a rule. The affinity depends not only on structural-functional features of synthetic peptides and full-length signal proteins but also on the physical-chemical properties of the medium (pH, salts, polar and non-polar solvents, etc.), the presence of hydrophobic surfaces, microenvironment of signal proteins, and other factors. 

## 11. Conclusion 

Many approaches are used now to study hormonal signaling systems and to develop specific and selective regulators controlling their functional activity; the peptide strategy, however, has some advantages compared to the others. First, the length of biologically active signal protein-derived peptides does not usually precede 15–20 amino acid residues, and they can be synthesized in a short time and in *quantum satis*, using the solid-phase method, purified to homogeneity by reversed phase HPLC and characterized by mass-spectrometry, amino acid analysis, and other appropriate methods. Second, to enhance biological activity and selectivity of the action of peptides and to obtain their analogs with higher stability to hydrolytic enzymes, signal protein-derived peptides are subjected to different modifications, such as selective blocking of free functional groups of amino acids, substitution of L- by D-amino acids and of natural amino acids by rare, nonnatural, amino acids, synthesis of cyclic and branched forms of peptides and their di- and oligomeric constrains, and attachment to them of hydrophobic radicals. Third, to obtain cell-penetrating forms of peptides, the latter can be cross-linked either with the MPS represented by short polycationic segment containing five or more arginine residues, the amphiphilic helix having hydrophilic positively charged side with lysine residues, often-applicable sequence 48–60 of HIV-1 TAT protein and signal sequence of Kaposi fibroblast growth factor, or modified by the hydrophobic moiety such as hydrophobic TM helical segment, hydrophobic fatty acid, and steroid radicals mimicking TM of intact signal protein. In the recent years in the synthesis of peptides wide application has found the minigene approach, a new biotechnology method in the frame of the peptide strategy. This approach is very perspective for the synthesis of very long peptides corresponding to full-length subdomains of signal proteins as well as of chimeric constrains including combination of regions derived from different signal proteins, but it is not applicable for obtaining modified by D- and nonnatural amino acids, branched and oligomeric as well as cell-penetrating forms of the signal protein-derived peptides, and cannot be used with all cell types. At the same time, a further modification of the minigene approach with the aim to expand its applicability must go on, especially because it allows creation of a whole library of signal-protein-derived peptides for deciphering a complex network of intracellular signal cascades and their selective regulation and modulation. It is of special importance that the minigene approach can be used to control the function of the central nervous system, where small minigene-expressing peptides may serve as selective intracellular regulators and modulators of neuronal signal transmission. 

It should be said in conclusion that on the addition to peptides derived from signal proteins, the components of hormone-sensitive signaling systems, some peptides generated from nonsignal proteins are also able to selectively regulate and modulate cell signaling, acting as a kind of bioactive molecules within cells [180]. These peptides are released in large amount by proteasomes and other extralysosomal proteolytic systems and before complete degradation can interfere with signal protein interaction, thereby affecting signal transduction, gene regulation, metabolism, protein targeting, and apoptosis. In pathological conditions a change in the composition of these intracellular nonsignal protein-derived peptides can have influence on the activity of hormonal signaling systems, especially their downstream components, and lead to disturbances in cell homeostasis. 

## Figures and Tables

**Table 1:** The peptides, derivatives of signal proteins, and their biological activity.

Signal protein	Sequence	Activity	References
*α*_2_-AR (C-ICL3)	RWRGRQNREKRFTC^361-373^ and its dimeric constrain with N-ICL3-peptide RIYQIAKRRTR^233-243^	Both selectively stimulate G_i/o_ proteins and inhibit *α*_2_-AR agonist-stimulated GTPase activity	[30–32]

*β*_2_-AR (C-ICL3)	RRSSKFCLKEKKALK^259-273^	Selectively stimulates G_s_ proteins and decreases regulatory effects of *β*_2_-AR agonists	[33–35]

D_1_-DR (C-ICL3, I), 5-HT_6_R (C-ICL3, II)	FKMSFKRETKVLKTLSV^260-276^ (I); KHSRKALKASL^258-268^K (II)	Stimulate G_s_ proteins and AC activity, decrease the stimulating effects of D_1_-DR (peptide I) and 5-HT_6_R (II) on AC system	[38, 39]

D_2_-DR (N-ICL3, I), 5-HT_1A_R (N-ICL3, II); 5-HT_1B_R (C-ICL3, III); 5-HT_1D_R (N-ICL3, IV; C-ICL3, V); m_4_-MChR (C-ICL3, VI)	VYIKIYIVLRRRRKRVNTK^206-224^ (I); LYGRIFRAARFRIRKTVK^214-231^ (II); ARERKATKTL^307-316^ (III); LYGRIYVAARSRI^213-225^ (IV); RKRISAARERKATK^285-298^ (V); RNQVRKKRQMAARERKVTR^382-400^ (VI)	Selectively activate G_i/o_ proteins and inhibit forskolin-stimulated AC activity in the absence of hormone; inhibit signaling via their cognate G_i/o_-coupled receptors	[30, 32, 39–42]

m_3_-MChR (C-ICL3)	LVKEKKAAQTLSAILL^483-497^	Selectively activates G_q_ and influences m_3_-MChR-mediated signaling	[43]

*δ*-opioid receptor (ICL3)	MGRLRSVRLLSGSKEKDRSLRRITR^237-261^	Blocks *δ*-agonist-induced PLC activation, Ca^2+^ release and cAMP signaling	[45]

Luteinizing hormone receptor; FSH receptor (C-ICL3, II); relaxin receptor RXFP1 (C-ICL3, III); parathyroid hormone receptor (C-ICL3, IV)	FAVQNPELMATNKDTKIAKK^551-570^ (I), HIYLTVRNPNIVSSSSDTRIAKR^533-555^ (II), and EIRNQVKKE(Nle)ILAKR^615-629^ (III) and their short analogs; EYRKLLK^402-408^ (IV)	Activate preferably G_s_ proteins and stimulate the basal AC activity, inhibit agonist-induced signaling via their cognate receptors	[46–52]

GLP1R (ICL1 and ICL3)	FRHLHCTR^169-176^; IVIAKLKANLMCKTDIKCRLAK^330-351^	Activate preferably G_s_ and G_i_ proteins, inhibit hormone- stimulated AC activity	[53]

V_2_-vasopressin receptor (ICL3)	Cyclo-QVLIFREIHASLVPGPSERAG-RRRRGRRTGSPSEGAHVSAAMAKT-VRMT^225-273^	Decreases the affinity of agonist for the receptor and inhibits vasopressin-stimulated AC activity	[55]

CB_1_-cannabinoid receptor (N-ICL3, I; C-ICL3, II, and N-CTD, III)	KAHSHAVRMIQRGTQKS^301-317^ (I); QVTRPDQARMDIRLAK^329-344^ (II); RSKDLRHAFRSMFPSCE^401-417^ (III)	Selectively stimulate different isoforms of the *α*-subunits of G_i_ proteins and significantly inhibit AC activity	[56–58]

Angiotensin II receptor of the type 1 (ICL2, I; N-ICL3, II; C-ICL3, III, N-CTD, IV)	DRYLAIVHPMKSR^125-137^ (I); TLIWKALKKAYEIQKN^216-231^ (II); KNKPRNDDIFRI^230-241^ (III); FLGKKFKKYFLQL^304-316^ (IV)	Inhibit angiotensin-induced activation of G proteins and the effector enzymes; peptide II selectively activates G_i/o_ proteins, peptide III G_q/11_ proteins	[59–62]
FPR1 (ICL2, I; N-CTD, II)	CVLHPVWTQNHR^126-137^ (I); FRERLIHALPASLER^308-322^ (II)	Activate in a PTX-dependent manner G_i_ proteins; peptide II inhibits high affinity agonist binding to the receptor and its coupling to G_i_ protein	[63, 64]

Prostacyclin receptor (ICL1)	SARRPARPSAFAV^39-51^	Selectively stimulates G_s_ proteins and the basal activity of AC	[65, 66]

Insulin receptor (tyrosine kinase region)	N-stearyl-TRDIYETDYYRK^1142-1153^	Increases insulin- and vanadate-stimulated phosphorylation of IR; significantly enhances insulin-induced stimulation of PI3K and MAPK activities	[67]

Insulin receptor (C-terminal region)	N-stearyl-SSHCQREEAGGRDGG^1293-1307^	Enhances insulin-stimulated autophosphorylation; increases insulin-stimulated PI3K and MAPK activities	[68]

EGF receptor (N-terminal region of the juxtamembrane domain)	RRREIVRKRTLRR^646-658^	Activates G_s_ proteins, influences activity of G_i_ proteins	[69]

NPR-C (cytoplasmic domain)	KKYRITIERRNH^461-472^; RRNHQEESNIGK^469-480^; RRNHQEESNIGKHRELR^469-485^; HRELREDSIRSH^481-492^ and their analogs	Inhibit the basal, forskolin- and hormone-stimulated AC activity; selectively increase GTP binding activity of G_i1_ and G_i2_ proteins; stimulate PLC*β*3 activity; inhibit hormone-stimulated DNA synthesis in vascular smooth muscle cells; induce smooth muscle contraction	[70–72]

NPR-C (extreme C-terminal region of the cytoplasmic domain)	GKHRELREDSIRSHFSVA^479-496^	Inhibits ANP(4–23)-induced G_i_ protein activation and cellular responses acting as a competitive inhibitor of ANP(4–23)-mediated signaling	[71]

G*α*_t_	IKENLKDCGLF^340-350^	Stabilizes the active state of opsin and meta-II-rhodopsin and meta-Ib-rhodopsin;	[73–76]

G*α*_s_	RVFNDARDIIQRMHLRQYELL^374-394^ and its short analogs	Inhibit transduction of hormonal signal via G_s_-coupled receptors	[77]

G*α*_s_ (Switch I and II regions, resp.)	RCRVLTSGIFETKFQVDK^199-216^; FDVGGQRDERRKWIQ-CFNDVTAIIFV^222-247^	Increase the basal and forskolin-stimulated AC2 and AC6 activities; inhibit G*α*_s_-stimulated activity of both AC isoforms; behave as partial agonist	[78]

G*α*_s_ (*α*3-*β*5 region)	EALNLFKSIWNNRWL-RTIS^268-286^	Inhibits the basal and forskolin-stimulated activities of AC2 and AC6; significantly decreases the G*α*_s_-stimulated enzyme activity	[78]

AC1 (C_1b_ subdomain)	IKPAKRMKFKTVCYLLVQLMHCRKMF-KA^495-522^ (pAC28, C_1b_)	Binds CaM with high affinity in a Ca^2+^-independent manner; inhibits CaM-stimulated AC activity with IC_50_ equal to 500 nM	[79, 80]
AC1 (C_2a_ subdomain)	TEEVHRLLRRGS-YRFVCRGKV^1024-1044^ (pVLG, C_2a_)	Binds CaM with low affinity in a Ca^2+^-dependent manner; inhibits CaM-stimulated AC activity with IC_50_ is 10 *μ*M	[80]

AC2 (C_2_ domain)	YTESDVNKEGLECLRLLNEIIADFD-DLL^899-926^ (*α*2, I); SKPKFSGVEKIKTIGSTYMAAT^927-948^ (*β*2-*β*3, II); DAINKHSFNDFKLRVGINHGPVIA-GVIGAQK^984-1015^ (*α*3-*β*4, III)	All peptides inhibit G*α*_s_- and forskolin-stimulated activities of AC2 and AC6; peptides I and III inhibit the basal AC activity; peptide I decreases Mn^2+^-stimulated activity	[81]

AC6 (C_1_ domain)	AAENHCLRIKILGDCYYC^427-444^ (*α*2-*β*2-*β*3, I); IHSGRVHCGVLGLRKWQFDVWSN-DV^487-511^ (*β*4-*β*5-*α*4, II)	Both peptides inhibit G*α*_s_- and forskolin-stimulated AC activities; peptide II inhibits the basal activity of both AC2 and AC6	[81]

PLC*β*1b	N-Myristoyl-TPPNPQALKW^1164-1173^ (I); GEGSSSVLSESCHEDPSVPPNFTPP-NPQALKW^1142-1173^ (II)	Both peptides dissociate the enzyme from membrane and inhibit PLC stimulation by hormones; peptide II prevents cardiomyocytes hypertrophy	[82, 83]

PLC*β*3	QEENTQL^1161-1167^	Significantly inhibits intracellular calcium response to selective agonists of mGluR	[84]

PLC*γ*	GLYRKAMRLRYPV^724-736^, and its N-myristoylated analogs	Specifically inhibit PLC activity and PLC-dependent cellular processes	[85]

PLC*δ*1 (IQ-peptide)	VRSQVQHKPKEDKLKLVPELS^473-493^	Inhibits PLC activity; binds CaM in Ca^2+^-independent manner	[86]

PLC*δ*1	N-Myristoyl-TIPWNSLKQGYRHVHLL^747-763^	Inhibits FSH-induced Ca^2+^ influx	[87]

Regulatory p85-subunit of PI3K (C-terminal region of N-terminal SH2 domain)	WNVGSSNRNKAENLLRGKR^11-29^	Exhibits binding specificity and affinity for PI 3,4,5-P_3_ and inhibits PI 3,4,5-P_3_-binding to the p85 subunit	[88]

PKC*ζ* (pseudosubstrate region)	N-Myristoyl-SIYRRGARRWRKL^114-126^	Inhibits PKC*ζ* activity; stimulates Akt, ERK1/2, p38 MAPK and eNOS activity	[89]

PKC*η* (pseudosubstrate region)	N-Myristoyl-RKRQRAMRRRVHQING^156-171^	Inhibits PKC*η* activity; stimulates eNOS phosphorylation	[89]

## Abbreviations

AC: Adenylyl cyclase
ANP: Atrial natriuretic peptide
AR: Adrenergic receptor
CaM: Calmodulin
CTD: C-terminal domain
DR: Dopamine receptor
EGF: Epidermal growth factor
FSH: Follicle-stimulating hormone
GLP1R: Glucagon-like peptide-1 receptor
GPCR: G protein-coupled receptor
5-HTR: 5-hydroxytryptamine receptors
ICL1, ICL2, and ICL3: The first, second and third intracellular loops;
IR: Insulin receptor
MAPK: Mitogen-activated protein kinase
MChR: Muscarinic acetylcholine receptor
mGluR: Metabotropic glutamate receptor
MPS: Membrane-permeable sequence
N-CTD: N-terminal region of CTD
N- and C-ICL3: N- and C-terminal regions of ICL3
NPR-C: Natriuretic peptide clearance receptor of C-type
PAR: Protease-activated receptor
PCI: PLC inhibitor
PI3-K: Phosphatidylinositol 3-kinase
PI 3,4-P_2_ and PI 4,5-P_2_: Phosphatidylinositol 3,4- and 4,5-diphosphates;
PI 1,4,5-P_3_ and PI 3,4,5-P_3_: Phosphatidylinositol 1,4,5- and 3,4,5-triphosphate
PKC: Protein kinase C
PLC and PLD: Phospholipases C and D
PT: Pertussis toxin
S1P: Sphingosine-1-phosphate
TM: Transmembrane region
TMC: Transmembrane channel
TXA_2_R: Thromboxane A_2_ receptor.
